# Detection and correction of artefacts in estimation of rare copy number variants and analysis of rare deletions in type 1 diabetes

**DOI:** 10.1093/hmg/ddu581

**Published:** 2014-11-25

**Authors:** Nicholas J. Cooper, Corina J. Shtir, Deborah J. Smyth, Hui Guo, Austin D. Swafford, Manuela Zanda, Matthew E. Hurles, Neil M. Walker, Vincent Plagnol, Jason D. Cooper, Joanna M.M. Howson, Oliver S. Burren, Suna Onengut-Gumuscu, Stephen S. Rich, John A. Todd

**Affiliations:** 1 JDRF/Wellcome Trust Diabetes and Inflammation Laboratory, Department ofMedical Genetics, NIHR Cambridge Biomedical Research Centre, Cambridge Institute for Medical Research, University of Cambridge, Wellcome Trust/MRC Building, Cambridge Biomedical Campus, Cambridge CB2 0XY, UK,; 2 Wellcome Trust Sanger Institute, Genome Campus, Hinxton, Cambridgeshire CB10 1SA, UK,; 3 University College London, Darwin Building, LondonWC1E 6BT, UK,; 4 Cardiovascular Epidemiology Unit, Department of Public Health and Primary Care, University of Cambridge, Worts Causeway, Cambridge CB1 8RN, UK; 5 Center for Public Health Genomics, West Complex, University of Virginia, Charlottesville, VA 22908, USA

## Abstract

Copy number variants (CNVs) have been proposed as a possible source of ‘missing heritability’ in complex human diseases. Two studies of type 1 diabetes (T1D) found null associations with common copy number polymorphisms, but CNVs of low frequency and high penetrance could still play a role. We used the Log-R-ratio intensity data from a dense single nucleotide polymorphism (SNP) array, ImmunoChip, to detect rare CNV deletions (rDELs) and duplications (rDUPs) in 6808 T1D cases, 9954 controls and 2206 families with T1D-affected offspring. Initial analyses detected CNV associations. However, these were shown to be false-positive findings, failing replication with polymerase chain reaction. We developed a pipeline of quality control (QC) tests that were calibrated using systematic testing of sensitivity and specificity. The case–control odds ratios (OR) of CNV burden on T1D risk resulting from this QC pipeline converged on unity, suggesting no global frequency difference in rDELs or rDUPs. There was evidence that deletions could impact T1D risk for a small minority of cases, with enrichment for rDELs longer than 400 kb (OR = 1.57, *P* = 0.005). There were also 18 de novo rDELs detected in affected offspring but none for unaffected siblings (*P* = 0.03). No specific CNV regions showed robust evidence for association with T1D, although frequencies were lower than expected (most less than 0.1%), substantially reducing statistical power, which was examined in detail. We present an R-package, *plumbCNV*, which provides an automated approach for QC and detection of rare CNVs that can facilitate equivalent analyses of large-scale SNP array datasets.

## Introduction

Rare (<1% population frequency) copy number variants (CNVs), usually deletions or duplications of 100–500 kb and larger, potentially play an important role in human disease (1). CNVs account for more base pair differences between individual genomes than single nucleotide polymorphisms (SNPs) (2,3) and are at the forefront of evolution, providing genetic material that facilitates the acquisition of new functions and speciation. Whilst widespread associations (involving 15% of cases) with CNVs have been reported for neurodevelopmental disorders (4–10), and niche associations (from 0.4 to 4% of cases involved) for obesity (11–15) and congenital heart disease (16,17), there have been few conclusive studies for autoimmune diseases. In addition, the contribution of smaller (<100 kb) CNVs to any disease is largely unexplored. A barrier to the characterization of CNVs in the genome is the availability of affordable technology to reliably detect and call CNVs in large numbers of individuals, using comparative genomic hybridization (CGH) or SNP arrays. Recently, researchers designed the ImmunoChip (18) to densely genotype 186 loci that exhibited statistically robust association with 11 immune-modulated diseases. Use of the ImmunoChip in very large numbers of case, control and family samples provides the opportunity to test the association of rare and, owing to the high density of SNPs, shorter CNVs in these disorders.

Type 1 diabetes (T1D) exhibits strong clustering in families, and yet the major effect, human leukocyte antigen (HLA) gene polymorphisms in the major histocompatibility complex (MHC), and over 50 other loci mapped outside the MHC (19–28), do not fully explain the familial aggregation. Failure to fully account for the heritability of T1D could be in some part due to CNVs, although there are many other potential contributors, including (as yet) unmapped common variants (29), rare or infrequent single nucleotide variants (SNVs) that segregate within individual families (30), limited linkage disequilibrium (LD) between the associated SNP and the causal variants in the locus (25) or the combined effect of the associated variants with unique intra-familial environmental factors, such as transmission of microbiota from mother to child (31).

Compared with SNP-based association studies, there are very few published CNV association studies for autoimmune diseases, but early results suggested that this could be an important source of missing heritability (32–34). However, most of these studies were small and thus focused mainly on common CNVs (>5% frequency), also known as copy number polymorphisms (CNPs). These included psoriasis, *N* = 1075 (35); systemic lupus erythematosus, *N* = 1241 (36); Crohn's disease, *N* = 744 (37); Addison's disease, *N* = 705 (38); paediatric multiple sclerosis, *N* = 90 (39) and T1D, *N* = 60 (40), where in each instance, ‘*N*’ was the total number of cases and controls. The largest of these studies targeted rheumatoid arthritis, *N* = 5003 (41), and did report some associations with rare CNVs, and a global ‘burden’ whereby cases had twice the number of DELs as controls.

The Wellcome Trust Case Control Consortium (WTCCC) studied the contribution of common CNPs using CGH arrays (42). In 16 000 cases of eight common diseases (2000 cases for each disease, including T1D) and 3000 shared controls, CNPs were not found to be significant contributors to disease. Those CNPs that were shown to be associated with a disease did not confer higher risks than the individual common SNPs in strong LD with the neighbouring CNPs. In particular, 79% of CNPs in the WTCCC were strongly correlated (*r*^2^ > 0.8) with at least one SNP genotyped by the HapMap project (www.hapmap.org), suggesting that the effect of CNP variation would have been identified through SNP-based genome-wide association study (GWAS) as described previously (20).

The remaining CNPs not in high LD with SNPs have recently been explored (43). A custom CGH array targeted 4309 CNPs and found no association with T1D using the same Type 1 Diabetes Genetics Consortium (T1DGC) families utilized in this study.

Through these two studies, the effect of (common) CNPs, both tagged and untagged by SNPs, were found not to contribute significantly to missing heritability for T1D. It should be noted, however, that only 22% of structural variants with frequency <5% had *r*^2^ > 0.8 with at least one SNP (42). This suggests that rare CNVs, and CNVs not tagged by SNPs, may represent a source of genetic variation that could account for additional T1D risk.

Both studies (42,43) have also highlighted the difficulties of scoring CNPs and CNVs, where batch effects make unbiased evaluation of associations challenging (44,45). In order to overcome the daunting task of CNV detection, validation, and reduction of bias, we developed a pipeline of Log-R-ratio (LRR) quality control (QC) procedures to reduce the number of artefactual CNV calls. These QC steps can be applied to any complex phenotype and any SNP array data for the purpose of filtering out samples with problematic hybridization intensity yet otherwise good genotype call rates, for identifying and correcting technically induced batch effects before using the intensity data to call CNVs, and for further using the relationship between rare CNV calls and hybridization QC metrics to isolate difficult to detect plate effects.

In this study, we investigated rare CNVs (frequency <1%) in deoxyribonucleic acid (DNA) samples from 6808 T1D cases, 9954 controls and 2206 families genotyped with ImmunoChip, which is one of the largest CNV studies to date using a single array platform. The raw probe intensities used for genotyping were re-purposed to calculate LRR and beta-allele frequency (BAF) at each SNP location. These two parameters were utilized jointly to call CNVs using *PennCNV*. The sensitivity of the results to many alternative permutations of QC was assessed using *plumbCNV* to derive a final convergent CNV set. These rare deletions (rDELs) and rare duplications (rDUPs) were tested for overall ImmunoChip-wide ‘burden’ for cases versus controls, and for burden across three subsets of segment length. There were 383 copy number variation regions (CNVRs) tested for rare but highly penetrant T1D associations, but none were significant after correction for multiple comparisons. A power analysis is provided to show recommended sample sizes for a range of CNV frequencies and possible effect sizes.

## Results

### Data quality control

Extensive testing and configuration of QC procedures were conducted on this dataset, as detailed in the ‘Materials and Methods’ section and Supplementary Material, Methods S1–S11. After all steps of the pipeline were applied to the case–control dataset, 79.7% SNPs passed QC thresholds. Sample failure rates were modest, with 6.7% of 9998 controls and 5.3% of 6808 T1D cases excluded for low quality data. For the family dataset, 82.8% of SNPs passed QC. The sample failure rate was slightly higher in the family dataset, with 9.9% of 6451 unaffected and 9.3% of 6490 affected family members failing QC. Further detail on exclusions, distributions and thresholds can be found in Supplementary Material, Tables S1–S3.

### Testing the sensitivity of the QC pipeline to thresholds and criteria

A wide range of QC procedures were utilized for this study. To attempt to make the QC thresholds applied less arbitrary, and to obtain a fair balance between sensitivity (detecting true CNVs) and specificity (avoiding false-positive CNV calls), we undertook an extensive threshold calibration exercise. We conducted an experiment whereby the QC pipeline and subsequent CNV calls were tested for 54 different configurations of QC parameters (Supplementary Material, Table S5 and Fig. S1). The resulting sets of CNVs produced sometimes varied widely. Sensitivity and specificity were defined by comparison to CNVs from the database of genomic variants (DGV) that intersected ImmunoChip regions. Average quality scores and case–control burden odds ratios (ORs) were calculated for each run to assess the sensitivity to QC. There was a marked trend that less stringent configurations produced large positive burden ratios (for instance, between 1.2 and 3.0), while more stringent configurations led to an overall burden close to unity, and higher quality scores.

Subsequent analyses for case–control and family datasets were performed using the set of parameters that produced the best combination of sensitivity, validation and average CNV quality score. This comprised the ‘medium’ setting for both SNP and sample QC, the largest number of components for principal components (PC) correction and utilized all of the CNV-QC filters (corresponding to run 20 from Supplementary Material, Table S5). Independent estimates of the sensitivity and specificity for this configuration are presented in Supplementary Material, Methods S10, which compare well to consistency rates determined for *PennCNV* in a comparative study of CNV detection methods (46).

### T1D association analyses

**Figure 1.**
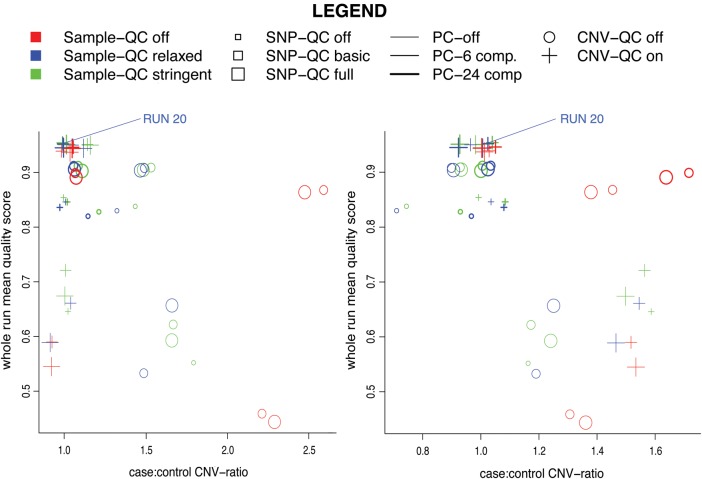
Quality score versus global burden ratio. From 54 runs of the pipeline with systematically differing QC configurations, this figure shows quality scores (average confidence percentage) versus global case–control ratio of the resulting set of CNVs called for rDELs (left) and rDUPs (right), respectively. See detailed legend for the meanings of the colour (Sample QC), size (SNP-QC), thickness (PC-correction) and shape (CNV-QC) of the points in the plot. It can be seen that there is an association of higher quality scores with a burden ratio nearer to 1.0. PC-correction (line thickness) has the largest magnitude of effect. All comparisons were made using rDELs/rDUPs inferred by at least six SNP sites. The Supplementary Material, Methods S3 contains information on how the number of sites we used was chosen.

There was no evidence for an overall burden of CNVs associated with T1D. Testing with different QC thresholds clearly showed a convergence to a ratio near 1.0 for the case–control rate of both rDELs and rDUPs (Fig. 1). For the subset of 54 runs of the *plumbCNV* pipeline with the ten highest average quality scores, the OR of case–control CNV burden ranged from 0.98 to 1.16 for rDELs (median 1.04), and 0.94 to 1.13 for rDUPs (median 1.05). This null result, and near to neutral OR, also held true when considering various CNV subsets, including (a) CNVs intersecting a gene, (b) CNVs intersecting an exon and (c) CNVs intersecting the DGV.

### Burden analysis by CNV length

We tested the burden of CNVs within three independent length subsets to determine if a significant enrichment in large CNVs could be detected in T1D cases (Table 1) and to search for systematic case–control frequency differences in CNVs too small to be detected by GWAS arrays.

**Table 1.** CNV burden by length analysis

Length	Controls	Cases	Odds-ratio	95% CI	FET-*P*-value
(A) rDELs					
0–20 kb	820	570	0.98	0.88, 1.10	0.7756
20–400 kb	514	372	1.03	0.89, 1.18	0.7256
>400 kb	74	82	1.57	1.14, 2.19	0.0053
(B) rDUPs					
0–50 kb	1098	740	0.95	0.86, 1.05	0.3017
50–400 kb	1336	946	1.00	0.92, 1.10	0.9451
>400 kb	326	230	1.00	0.84, 1.19	1.0000

An analysis of burden for rDELs (A) and rDUPs (B) across three subsets of CNV length. Each row contains results where the CNV set is filtered to include CNVs within the size window indicated (‘Length’). A Bonferroni threshold based on six tests was applied. No rDUP results were significant whereas the rDEL result for CNVs longer than 400 kb passed this threshold. Supplementary Material, Methods S11 shows no burden for the same length-subset analysis on the UVA (control) versus Sanger (control) groups. The threshold between the middle and small CNV sizes is 50 kb instead of 20 kb for rDUPs to account for the differing length distributions. There were not nearly as many rDUPs <20 kb (∼60% less), which would have made the middle category much larger than the first category. Locations used hg18 coordinates.

Three thresholds were chosen to reflect categories:

- *Small CNVs* (0–20 kb for rDELs, and 0–50 kb for rDUPs) with a different breakpoint in rDUPs due to very different length distributions for the smaller band between rDELs and rDUPs.
- *Medium CNVs* (20–400 kb for rDELs, and 50–400 kb for rDUPs), detectable by ImmunoChip and GWAS chips, with few reported associations to date.
- *Large CNVs* (greater than 400 kb) such as have been previously been associated with neurodevelopmental disorders.

There were a greater proportion of longer rDELs in T1D cases, OR = 1.57 (1.14–2.19), *P* = 0.005, but there was no difference for medium length or shorter rDELs. There were no differences within any length band for rDUPs.

See Supplementary Material, Methods S12 for a detailed description of the case and control distributions of CNV length.

### Burden estimate for the family dataset

A burden estimate was also calculated using the family data, by using the ratio of CNV transmission rates from Table 2, for affected versus unaffected children. Consistent with the case–control overall burden results, there was no difference between T1D and unaffected children, with a ratio of 1.01 for rDELs (44.2/43.8%) and 0.91 for rDUPs (42.3/46.3%). Transmission rates were somewhat lower than the theoretical expected rate of 50%, reflecting imperfect sensitivity and specificity of CNV calls.

**Table 2.** Transmission rates to affected versus unaffected siblings

Transmission	Affected (%)	Unaffected (%)	Ratio	*P*-value
DELs	44.2	43.8	1.01	0.90
DUPs	42.3	46.4	0.91	0.14

Analysis of transmission rates for CNVs within family trios, from parents to affected and unaffected children. For transmission percentages the denominator is the total number of possible transmissions for that category (i.e. the number of children multiplied by number of CNV-carrying parents in each family, summed across all CNVs). Additional data for de novo and transmission rates can be found in Supplementary Material, Methods S9 and S10. Note that there are roughly four times more affected than unaffected children in the family dataset. Prior to generating these percentages, CNVs were excluded from CNVRs that did not have at least one CNV with a quality score above 90%.

### Burden of de novo CNVs

Previous association studies utilizing family data have found that de novo CNVs are more likely to be deleterious as they have not been subjected to selection (16,47,48). There were 18 de novo rDELs observed amongst the T1D-affected offspring, but none in the unaffected siblings cohort (*P* = 0.03, *N*_aff_ = 5077, *N*_unaff_ = 1282). These de novo rDELs are presented in Supplementary Material, Table S6 alongside historical information and HLA classical types. The LRR and BAF intensity at each CNV locus were visually verified against any siblings, and both parents, to ensure that these were true denovos and not due to lack of sensitivity in detecting the same CNV in a parent. There was insufficient power to explore de novo rates in any sort of stratified manner. Regarding duplications, there were only eight de novo rDUPS detected in affected children, and five in unaffected, which did not comprise a statistically significant difference.

### ImmunoChip-wide CNVR analysis

This approach pools sets of overlapping CNVs, so that CNVs of slightly different lengths and positions can be compared as part of the same CNVR, and facilitates case–control testing of specific CNV loci. Significance values were generated using Fisher's Exact Test (FET) given the rarity of these events. Separate QQ-plots for rDELs and rDUPs show that *P*-values closely follow a uniform distribution suggesting no inflation of test-statistics (Supplementary Material, Fig. S3). Counts from 0 to 150 CNVs per region were observed from a total of 6524 T1D cases and 9238 control samples that passed QC.

CNVRs for rDELs and rDUPs were assessed across the ImmunoChip and the five most significant associations with T1D for each are reported in Table 3. None were significant after a Bonferroni correction for 383 regional tests (threshold *P* = 1.31 × 10^−4^).

**Table 3.** Top five FET results for CNVRs

Locus	Start (Mb)	End (Mb)	Length (kb)	Cases	Controls	OR	*P*	Genes
(A) rDELs								
8q21.12	79.65	79.67	22.9	6	1	8.59	0.0215	*PKIA*
6p21.31	34.75	34.76	6.1	4	0		0.0282	*C6orf106*
15q13.2-3	28.74	30.20	1461.0	4	0		0.0282	*TRPM1* + 6
6p21.31-32	33.60	33.63	29.0	15	8	2.69	0.0314	Intergenic
11q23.2	113.97	114.05	77.7	71	71	1.43	0.0321	Intergenic
(B) rDUPs								
14q13.2	34.55	34.82	267.7	0	8		0.0247	*SRP54* + 3
3p26.3	1.31	3.17	1862.3	4	0		0.0282	*CNTN6* + 5
10p15.2-3	2.00	3.20	1195.0	7	2	5.01	0.0376	*PFKP* + 1
17p12	14.13	15.32	1197.6	5	1	7.16	0.0457	*PMP22* + 4
16p11.2	28.39	28.93	540.1	0	7		0.0464	*SH2B1* + 14

The top five most statistically significant rDEL (A) and rDUP (B) results from an analysis of CNVRs. Region boundaries were defined using the Plink command ‘segment-group’. None of these results passed the Bonferroni corrected threshold of ∼*P* < 5 × 10^−5^. Genes are those overlapped by the bounds of the CNVRs. These counts have been filtered to include only CNVs passing quality score thresholds, and CNVRs with greater than 20% of CNVs failing on quality scores were excluded. The notation ‘+*n*’ in the genes column indicates the number of overlapping additional genes not listed. OR, odds-ratios, some of which cannot be calculated due to zero counts. Locations used hg18 coordinates.

In order to validate key output from our pipeline using an independent lab-based method, two of the rDELs from these top five associations, *PKIA*, and a region within 16p11.2 (encompassing 15 genes including *SH2B1*), were tested and successfully replicated with quantitative polymerase chain reaction (qPCR) (see, Supplementary Material, Table S7 and Methods S8). *PKIA* is involved in pancreatic beta-cell function (49) and 16p11.2 has been widely reported in the literature as contributing to the genetic risk of obesity (11–15), potentially linked to insulin signalling (50).

Because CNVs at 16p11.2 have been widely studied we were able compare the population frequencies we observed with several large studies (51,52). In our control samples, the 16p11.2 rDUP was detected with 0.07% frequency, which closely compares with a rate of 0.05% pooled from seven studies comprising nearly 60 000, Icelandic and Northern European controls (51). The same meta-analysis showed 0.04% incidence of 16p11.2 rDELs, similar to the observed rate of 0.03% in our control cohort. For both rDELs and rDUPs, the incidence in our controls matched very closely, providing a positive control for the sensitivity of our pipeline to detect very rare CNVs.

### TDT analysis of the family data

None of the transmission disequilibrium test (TDT) results for CNVRs passed the Bonferroni corrected threshold of *P* < 10^−4^. The results with the lowest *P*-values are presented in Table 4 for reference, but should not be considered as candidates for association without follow-up in a much larger sample. The chromosome 10q21.1 rDEL result seems merely to reflect that the CNV was detected with lowered sensitivity, as both cases and controls had a low transmission rate (close to 33%). The chromosome 6p22.1 rDEL and 6p21.33 rDUP both had transmission above 50% in controls and low transmission in T1D cases, consistent with being protective. Whilst these results are inconclusive due to lack of power, these two CNVs are located in the MHC (the region most associated with T1D) and could plausibly disrupt regulatory elements controlling expression of other MHC genes.

**Table 4.** Trios TDT test for CNVRs

Locus	Start (Mb)	End (Mb)	Length (kb)	Genes	Transmissions		TDT (*P*)
					Cases	Controls	
(A) rDELs							
10q21.1	59.76	59.76	4.0	Intergenic	28/81	10/29	0.0079
6p22.1	29.20	29.27	72.6	*OR2J4P* + 3	5/22	4/6	0.0253
12q12	39.06	39.10	39.5	Intergenic	5/5	1/2	0.0253
(B) rDUPs							
6p21.33	31.47	31.56	93.4	*MICA* + 4	9/38	5/7	0.0041

TDT analysis of CNV transmission for trios where at least one parent has a CNV and at least one child is affected, for rDELs (A) and rDUPs (B). Genes are those overlapped by the bounds of the CNVRs. In the ‘Genes' column, ‘+*n*’ indicates that in addition to the gene(s) indicated a further ‘*n*’ genes overlap that CNVR. The full listing for 6p22.1 was: *OR2J4P*, *SAR1P1* and *OR2B3*; and for 6p21.33 was*: MICA, HCP5, HLA-S, HLA-C* and *RPL3P2*. For transmissions, each cell contains a count of how many times a CNV was transmitted from a parent versus the total number of children of parents with that CNV. Note that in contrast to a SNP-based TDT analysis where it is customary to test against an implicit transmission rate of 50%, because the sensitivity of CNV detection can be different across the array, the transmission rate for controls should also be taken into account. Any CNV where controls had a significant TDT result in the same direction (suggesting under- or over-sensitivity) should be interpreted with caution (e.g. the rDEL in 10q21.1 above). These counts have been filtered to include only CNVs passing quality score thresholds. Locations used hg18 coordinates.

### Individuals with large CNVs

Very long CNVs are thought to be highly deleterious and likely to disrupt health. There is a high prior probability that such a CNV could contribute to the development of a disease state should it occur in a critical genomic region. Thus, a very long CNV in a region containing immune response genes could affect risk of T1D. For rDELs, eleven cases and only one control had a CNV greater than 3 Mb. While this length threshold was not part of planned testing, this yielded an OR estimate of 15.60 (95% CI = 2.27, 669.48; *P* = 0.00045).

CNVs of this length have been associated with developmental disorders, so this raises a question about sample selection and comorbidity. There was no difference in exclusion criteria between cases and controls. However, a slight bias could foreseeably have arisen due to self-selection. Given that most controls were adult blood donor volunteers they are unlikely to have an incapacitating illness or they would not have gone to the trouble. In contrast, child volunteers were brought in by their parents to contribute to T1D research, so could potentially have other illnesses comorbid with T1D.

All CNVs of this size or larger are presented below for the case–control and family datasets (Table 5). In the case–control dataset, it is unknown whether the long CNVs were truly de novo as parental data were unavailable. Both of the rDELs for T1D cases in the family data were confirmed as de novo. The repeated rDUPs in the families dataset on chromosomes four and nine each occurred within a single family. Given the small number of occurrences of each CNV, no statistical analysis could be performed; however, these exceptional genomic features are catalogued here as a reference for future work.

**Table 5.** Large CNVs

Locus	Start (Mb)	End (Mb)	Length (Mb)	Genes	Phenotype	SNPs
(A) Case–control dataset						
rDELs						
2p21	40.15	43.30	3.15	*SLC8A1* + 9	T1D	250
2q37.3	238.67	242.44	3.77	*SCLY* + *45*	T1D	288
3p11.2	83.01	87.41	4.40	*CADM2* + 3	T1D	38
3q28-29	193.33	196.62	3.29	*FGF12* + *15*	T1D	34
4q12	58.53	66.53	8.00	*LPHN3*, *EPHA5*	T1D	83
6q16.1	97.55	121.05	23.50	*KLHL32* + 112	T1D	1659
10q11.22-23	47.99	51.29	3.30	*ARHGAP22* + *40*	Control	650
10q22.3	81.64	88.94	7.30	*ZNF488* + 34	T1D	136
11q24.3	129.25	134.23	4.98	*KCNJ5* + 25	T1D	100
14q31.1	79.64	83.72	4.08	*DIO2* + 6	T1D	1276
18p11.31	0.17	5.76	5.59	*USP14* + 22	T1D	103
18q22.3-23	71.65	76.12	4.47	*ZNF516* + *13*	T1D	85
rDUPs						
3p21.1-21.31	48.33	52.85	4.52	*ZNF589* + 133	T1D	2636
4q12	54.18	57.77	3.59	*CHIC2* + 23	T1D	47
6p24.3-25.3	0.15	7.74	7.59	*DUSP22* + 45	T1D	407
8p23.1-3	1.66	9.85	8.19	*DLGAP2* + 77	Control	250
8p23.2-3	1.66	5.43	3.77	*DLGAP2* + 11	T1D	152
10q11.23-21.1	52.48	56.80	4.32	*PRKG1* + 6	Control	162
11p15.4-5	0.80	8.99	8.19	*RPLP2* + 215	Control	467
13q21.32-33	65.26	72.02	6.76	*PCDH9* + 3	Control	106
(B) Family dataset						
rDELs						
2q36.3-37.7	227.56	242.67	15.11	*RHBDD1* + 146	T1D	1750
5q11.2-12.3	55.28	64.52	9.23	*IL6ST* + 31	T1D	256
5q21.1-2	98.83	103.28	4.45	*FAM174A* + 8	Control	1146
rDUPs						
1p22.2-3	87.09	90.59	3.50	*HS2ST1* + 15	T1D, son	53
2p15-16.1	58.24	61.85	3.61	*VRK2* + 13	Control, mother	1373
4p12-13	44.50	47.97	3.47	*GABRG1* + 12	T1D, daughter	279
4q31.23-35.2	150.88	190.98	40.10	*DCLK2* + 134	T1D, unknown	471
4q32.1-35.2	161.48	190.98	29.50	*FSTL5* + 87	Control, father	369
4q34.3	178.73	182.09	3.36		Control, mother	34
4q34.3	178.73	182.09	3.36		T1D, daughter	34
9q31.1-2	104.21	107.38	3.17	*CYLC2* + 16	Control, mother	43
9q31.1-2	104.21	107.38	3.17	*CYLC2* + 16	T1D, son	43
9q31.1-2	104.21	107.38	3.17	*CYLC2* + 16	Control, son	43
11q24.1-25	122.30	134.27	11.97	*C11orf63* + 94	Control, father	850

Tabulation of specific rDELs and rDUPs longer than 3 Mb for the case–control (A) and family (B) datasets, respectively. In the ‘Genes’ column, ‘+*n*’ indicates that in addition to the gene(s) indicated further ‘*n*’ genes overlap that CNV. In the ‘phenotype’ column ‘Control’ means the sample came from the unaffected (families) or control groups (case–control). The ‘SNPs’ column shows the number of ImmunoChip SNPs passing QC within the bounds of each CNV. Within the family data (B), the chromosome nine rDUPs were from samples in the same family, and so are the chromosome four rDUPs with no gene overlap. Note that 11 rDELs of 3 Mb or longer were found in T1D cases versus only 1 rDEL in controls. Within the family-rDELs, both occurrences in affected were confirmed to be de novo. All locations use UCSC hg18 coordinates.

### Power analysis

**Figure 2.**
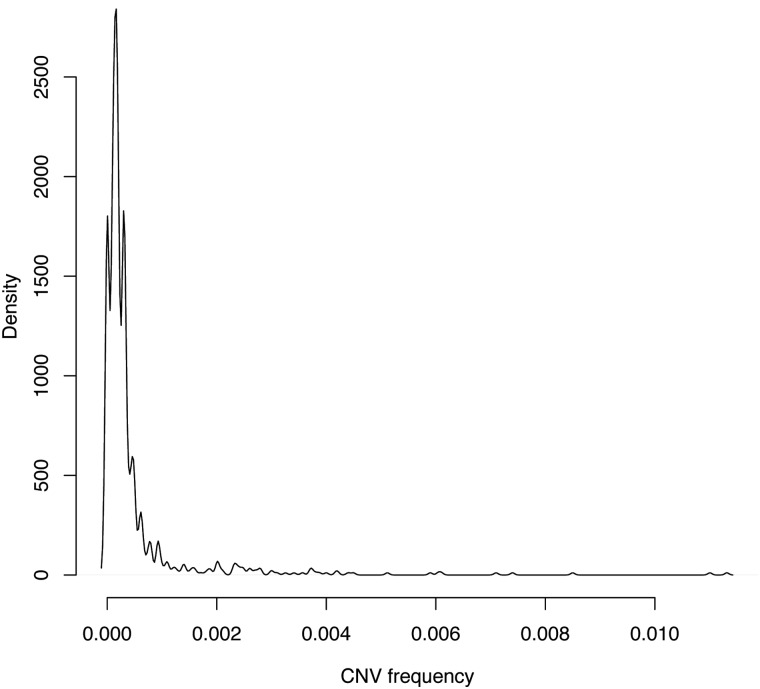
Density distribution of CNVRs. Density plot for CNV frequency for CNVRs in cases and controls. Note that most (rare) CNVs detected occurred at a frequency of <0.1%. Even when the *x*-axis was extended to include CNPs up to 5% frequency (this figure stops at 1%), this right-skewed distribution continued to be extremely sparse in the right hand tail (Supplementary Material, Fig. S4). This graph is calculated *per region*, so a CNVR with one sample gets the same weighting in this figure as a CNVR containing 100 samples. Supplementary Material, Figure S4 shows histograms calculated *per sample*, which gives a more uniform distribution.

This study had sufficient power for CNV burden analyses; however, the lack of specific regional hits may be attributed to limited power for multiple testing of every CNVR. Estimated total sample sizes required to conduct genome-wide associations for CNVRs are presented in Table 6, stratified by case–control ORs and CNV frequency. The current study case–control sample size is included as ‘1.6’ (i.e. 1.6 × 10 000 = 16 000), with results showing that we had sufficient power to detect CNVs with an OR of 2.0 at frequency 1%, an OR of 3.0 at frequency 0.5%, and that we would require an OR greater than 7.0 to detect CNVs less frequent than 0.2%. However, 91.6% of CNVRs were less frequent in our dataset than 0.1% (Fig. 2, and also Supplementary Material, Fig. S4 for more detail on the distributions of CNV frequency in this study). This power analysis suggests that to explore CNVs for association with disease with plausible effects sizes (for instance, an OR range of three to ten), a dataset of 30 000 cases and 30 000 controls would be required.

**Table 6.** Power analysis for genome-wise CNVR studies

CNV frequency	OR													
	20	10	9	8	7	6	5	4	3	2	1.75	1.5	1.25	1.1
0.0001	20	40	40	40	40	40	40	60	80	160	400	600	1400	8000
0.0002	10	12	12	14	16	18	20	40	40	80	120	400	800	4000
0.0005	4	6	6	6	8	8	8	10	16	40	60	100	400	1600
0.001	2	4	4	4	4	4	4	6	8	16	40	60	140	800
0.002	1	1.6	1.6	1.6	1.6	2	2	4	4	8	12	40	80	400
0.005	1	1	1	1	1	1	1	1	1.6	4	6	10	40	160
0.01	1	1	1	1	1	1	1	1	1	1.6	4	6	14	80
0.02	1	1	1	1	1	1	1	1	1	1	1.6	4	8	40
0.05	1	1	1	1	1	1	1	1	1	1	1	1	4	16

Power analysis of the approximate total case + control sample size (in units of 10 000 samples) required to pass the Bonferroni *P*-value threshold for a genome-wide CNVR analysis using ImmunoChip for different case–control ORs and CNV frequencies. The expectation is that the number of CNVR regions called using the Plink method would saturate to ∼1000, so the Bonferroni threshold used was 5 × 10^−5^. The last two frequency rows are technically CNPs (common CNVs) as defined in this study.

## Discussion

Several studies of T1D have found null associations with CNPs (42,43), but CNVs of low frequency and large effect could still play a role. There is strong evidence for the role of large rare CNVs exceeding 400 kb, contributing to risk of neurodevelopment disorders (7,8,47,53–55). Results from a recent genome-wide CNV study of morbidity of development delay and those from 15 other neurodevelopment studies estimated that 15–20% of patients could be diagnosed with a structural abnormality (54). To determine the role of CNVs in T1D, we conducted both overall burden and locus-based tests, within a case–control and an independent family trios dataset, in one of the largest CNV studies to date for a common phenotype and methodology. Our QC pipeline was able to overcome significant biases in the LRR data, correcting for intensity differences in datasets originating from separate collections and sample types that differed systematically between phenotypes.

Because of the low frequency and heterogeneity of CNVs, one of the most important statistical tests in previous studies has been the burden test. By pooling CNVs together it provides an analyzable metric when locus specific counts are too small to calculate meaningful statistics. The burden ratio is clearly a crude statistic and is prone to misuse. First, any systematic batch effects that bias the intensity for cases or controls can easily manifest itself in the burden ratio, which we observed for test runs when PC correction was not applied to the LRR data. In fact, we found that an overall burden close to one seemed to be an excellent indicator for high quality CNV calls that showed high validation performance across multiple criteria. As a negative control, we were also able to demonstrate that burden tests on our two separately genotyped control cohorts converged to 1.0 (Supplementary Material, Methods S11). We would suggest that analysts of CNV datasets greet large overall burden values with scepticism, and to attempt to falsify possibly misleading results by systematically tuning QC procedures.

Second, a burden test assumes not only an increased rate of CNVs, but that the directions of associations are not balanced; equivalent to saying that most deletions (DELs) would increase the likelihood of disease, whereas it may be that a harmful locus could be suppressed as a result of an rDEL. Hypothetically, an equal number of protective CNVs in controls and harmful CNVs in cases could be hidden in a neutral burden estimate. This could be true of the current study, but a larger dataset with power to conduct CNVR tests at very low frequencies and moderate effects sizes would be required to test for this.

Third, there are some conceptual issues with the interpretation of a large global difference in genome-wide CNVs between two cohorts of similar ethnic ancestry. CNVs can occur almost anywhere and most probably have little impact on health. More informative studies seek to conduct burden tests on subsets of CNVs with some meaning, so for instance: overlapping gene pathways previously associated with the study phenotype, overlapping genes predicted as haploinsufficient, overlapping coding sequence or epigenetic features. Because this study used ImmunoChip, the CNV set was already enriched for autoimmune regions, providing an interpretation for our burden test. To explore potential enrichment in further detail, we also examined de novo CNVs in the family data, and subsets of CNV length, as longer CNVs are more likely to have functional consequences.

We found a strong null result with respect to the overall burden of rDUPs with a case–control ratio near 1.0, even when filtering by genic or exonic overlap, and across three CNV length groups. This result may be generalizable and could suggest that, for T1D risk, additional copies do not result in increased or modified expression for the majority of genes.

We did obtain evidence for an increased burden of rDELs with T1D risk. Those greater than 400 kb in size were found to be over 50% more frequent in cases. For very large CNVs (greater than 3 Mb); 11 were observed in cases, and only one in controls. These data suggest that loss of function in the immune-enriched regions of the genome contribute to T1D risk. Longer rDELs are likely to disrupt a greater number of genes, and thus have more chance to intersect genes in immune-pathways that are haploinsufficient (48), requiring two copies for normal function. Longer CNVs are also more likely to be de novo (45) and thus have not undergone negative selection against deleterious function.

Importantly, rDELs of this size were rare, applying to only 1.26% of T1D cases (*n* = 84) and 0.80% of controls (*n* = 74). This effect is much smaller than observed in neurodevelopmental disorders, even when taking into account that those estimates are based on GWAS arrays, which have 39% greater coverage of the genome for CNVs of this size (see coverage comparison in Supplementary Material, Table S8).

A key advantage of the ImmunoChip over GWAS arrays for CNV detection is the ability of the ImmunoChip to target smaller CNVs in immune regions. The smaller CNVs formed a large portion of the CNVs detected: 57.2% of rDELs detected were <20 kb in length while 38.6% of rDUPs were <50 kb. In comparison, GWAS arrays detect CNVs smaller than 20 kb in T1D regions at roughly one quarter the rate of ImmunoChip. No evidence for burden of T1D risk was found for CNVs between 20 and 400 kb, or CNVs shorter than 20 kb in the case–control dataset.

In the family dataset, we observed 18 de novo rDELs in affected versus zero in unaffected samples. With such a low count, with only 0.3% of T1D samples implicated, and the likelihood that some of these rDELs have no effect on diabetes risk, we would conclude that de novo CNVs occurring in mapped autoimmune disease regions probably only affect a small minority of T1D cases.

The lack of regional CNVR association with T1D is not necessarily due to the absence of genuine association but partially to the ‘rareness' of rare CNVs. Despite setting the frequency threshold at 1%, the vast majority (>91%) of CNVs were ten times more rare than this (<0.1%). This observation is consistent with two recent studies of SNVs and rare mutations using exome sequencing, which found that 86% of coding variation had frequency <0.5%, the ratio of rare to common SNVs was six to one (56) and that 56% of CNVs in a dataset of ∼5000 samples were singletons (57). In the case–control dataset for this study, more than 75% of CNVs were singletons. This evidence for a frequency ‘trough’ between rare and common variant frequencies (between 0.1 and 5%) is entirely consistent with purifying selection, and findings that CNPs do not generally associate with disease (42,43); if they did, they should have already been selected against. Further, in the SNV study above, the predicted deleteriousness for variants below 0.5% frequency was 4.2 times that of more common variants (56).

These very low counts for CNVRs resulted in low statistical power where only an extreme case–control ratio could exceed the critical value (adjusted for multiple testing). We did observe some CNVRs with ORs up to eight. If these ratios remained consistent, our power analysis (Table 6) suggests that a sample size of 30 000 cases and 30 000 controls would be required to pass multiple testing correction. The low cost of genotyping arrays indicates that such a sample size could be examined in a cost-effective manner in the near future.

The critical aspects of data cleaning and QC identified in this study can overcome many of the barriers to testing for association with rare CNVs in complex human diseases. In conjunction with this analysis, we developed an R package, *plumbCNV* (58) that automates the QC and CNV-calling pipeline with extensive control of thresholds. The final set of CNVs detected in this study using this pipeline have been validated in multiple datasets, comparing common samples on MetaboChip, comparing CNVs to the DGV, examining transmission rates in trio data and by qPCR replication of specific loci. The software is fast, scalable and allows flexible input and verbose output. The package simplifies CNV analysis, facilitating validation and optimization of QC thresholds and we would encourage researchers with large ImmunoChip, MetaboChip or other custom genotyping array datasets, to obtain further value from their microarray datasets by exploring rare CNV associations with their phenotype.

Even with the discussed deficits in power and coverage, our findings suggest that rare CNVs could increase the burden of risk for T1D. The effect of rDELs on T1D would likely be heterogeneous and pertain to a small minority of patients, given the low frequency of variants longer than 400 kb, the rarity of de novo CNVs in autoimmune disease regions and the lack of case–control burden for CNVs shorter than 400 kb. A future association analysis of CNVRs in a larger dataset could help to identify specific regions that may give insights into the mechanistic understanding of T1D. Implicated loci could differ to those identified through SNP-based GWAS and may be easier to interpret because rDELs can ‘knock-out’ entire genomic features, rather than simply modifying selected alleles. Importantly, our results highlight the challenges of scoring CNVs in any disease and the necessary QC metrics required to assure robust statistical results.

## Materials and Methods

### Subjects

Samples for this study were derived from several sources (Table 7). Written informed consent was obtained from all subjects with approval from the ethics committee or institutional review board of all participating institutions. The cases in the case–control analysis were part of the UK GRID T1D case collection (UK GRID) (60), with cell-lined DNA samples as available from the National Institute of Diabetes and Digestive and Kidney Diseases (NIDDK) Central Repository (63). The controls in the case–control analysis were taken from the 1958 British Birth Cohort (1958BC) (64) and the United Kingdom Blood Services Common Controls (UKBS-CC) and are unselected population controls (60). The 1958BC samples were cell-lined DNA samples and are available from Bristol, UK (61); the UKBS-CC samples are genomic DNA and are available from Cambridge, UK (59). The average (median) age at diagnosis in cases was 7.7 (8.0), with first and third quartiles of 4.0 and 11.0. Finally, there was no bias in geographic distribution of cases and controls (65).

**Table 7.** Description of ImmunoChip datasets used for this paper

Study dataset	Samples	Phenotype	Typing centre	Sample-source	Sample type	MetaboChip data
Case–control	4537	Controls	Sanger	UKBS-CC	Genomic DNA	No
Case–control	6808	T1D	UVA	UK GRID	Cell line DNA	No
Case–control	5461	Controls	UVA	1958BC	Cell line DNA	Yes
Families	13 070	T1D and Families	NIDDK	T1DGC-ASP	Cell line DNA	No

Tabulation of the data sources used in this study. The two datasets referred to through are the study datasets ‘case–control’ and ‘families'. ‘T1D and families' encompasses T1D-affected and unaffected siblings and their parents. Sample sources are UKBS-CC (59); UK GRID (60); 1958BC (61) and T1DGC-ASP (62). A ‘yes' for the ‘MetaboChip data’ column indicates that in addition to the ImmunoChip dataset, there is a parallel dataset for the same cohort for the Illumina iSelect MetaboChip array.

Family data are independent of the case–control subjects and derived from the T1DGC collection (62). The T1DGC initiated an affected sib-pair collection with focused identification of families with two or more children with T1D, both parents available, and collection of an unaffected sibling when possible. As a result, over 80% of families have both parents available. Family samples are predominantly cell-lined DNA with some genomic samples and are available from the NIDDK Central Repository (63). The average (median) age at diagnosis in affected offspring was 10.7 (9.0), with first and third quartiles of 5.0 and 14.0. Amongst the T1D families, 4937 parents were unaffected and 199 were affected. Amongst the siblings, 6291 were affected and 1514 were unaffected.

A total of 201 plates with 96 samples per plate were used to type the data (although some plates were incomplete). Although the majority of samples were genotyped at one location (the University of Virginia, UVA), the case–control and family samples were genotyped at different times and, for controls, at different locations (UVA and the Wellcome Trust Sanger Institute (Sanger)). There were 6808 UVA-typed cases (84 plates), 5461 UVA-typed controls (genotyped on a different date to the T1D cases, 78 plates) and 4537 Sanger-typed controls (49 plates).

This diversity of sample sources presented a considerable obstacle for batch effects correction procedures. Pre-QC raw LRR data for 83.0% of all SNP sites passing QC showed significant (*P* < 3.14 × 10^−7^) intensity differences between cell line and genomic DNA, and 90.2% showed intensity differences between T1D and control phenotypes. The QC procedures implemented resulted in a reduction of these difference-rates to only 1.4% and 1.2%, respectively. This set of 1937 SNPs that still differed were excluded before calling CNVs in the case–control dataset.

### Platform

ImmunoChip is a consortium designed, custom Illumina iSelect two-colour bead-chip, targeted to fine map the genetics of autoimmune disease (18,66). ImmunoChip includes replication SNPs that had been associated with target diseases via GWAS at *P* < 5 × 10^8^, plus dense fine-mapping SNPs for the regions surrounding these loci, to facilitate search for causal variants. A total of 3000 fine-mapping SNPs were chosen for each of 12 diseases based on available GWAS data (rheumatoid arthritis, ankylosing spondylitis, systemic lupus erythematosus, T1D, autoimmune thyroid disease, coeliac disease, multiple sclerosis, ulcerative colitis, Crohn's disease, psoriasis, juvenile idiopathic arthritis and primary biliary cirrhosis). This non-uniform coverage presented an additional challenge versus similar analysis with GWAS arrays (Supplementary Material, Table S8).

Samples were genotyped using the ImmunoChip according to Illumina's protocols (UKBS-CC and 1435 of the 1958BC at the Sanger), Hinxton, UK; and UK GRID, 1958BC and T1DGC-ASP families at Charlottesville, VA, USA. Data are available from the European Genome-Phenome Archive (EGA) (59,61). NCBI build 36 (hg18) mapping was used throughout (Illumina manifest file, Immuno_BeadChip_11419691_B.bpm). The resulting locked ‘long-file’ format dataset containing LRR, BAF, genotypes and intensity information was the source data for the analyses to follow.

### Development of the CNV pipeline

CNV detection using SNP arrays is a complex undertaking, particularly in the presence of systematic batch effects. Initial naïve analyses where the raw data, with standard SNP-QC, were fed into pre-existing *PennCNV* software (67) yielded several seemingly promising CNV-loci and a strong positive case–control burden association. Individual CNVs examined could not be replicated. It soon became clear that extensive QC would be required to be able to detect genuine disease association signals.

The set of QC filters and PC-correction that comprise our recommended pipeline were established in conjunction with analysis of these T1D datasets, including qPCR validation of loci showing initial associations (Supplementary Material, Methods S8). Failure to replicate CNV calls with qPCR led to the development of QC procedures to address persistent biases. It should be noted that individually most of the QC steps described have been used before in other CNV studies (10,44,67,68 and Supplementary Material, Methods S4), although not in combination, or with as extensive calibration and validation.

**Figure 3.**
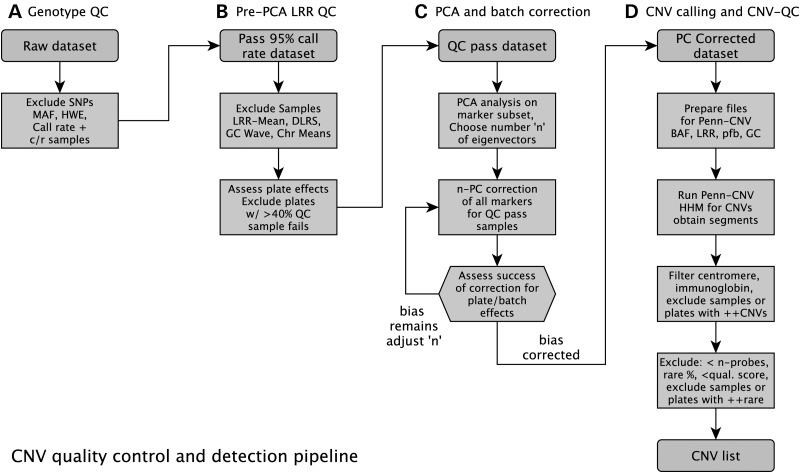
QC pipeline implemented in *plumbCNV*. Flow chart for our CNV detection pipeline, showing the QC steps involved. The rounded boxes are datasets and the hard-edged boxes are processes implemented by our custom R package, *plumbCNV*, currently available on github (58).

Once a set of core methods was in place (Fig. 3), the pipeline was automated using R scripts and 54 iterations (to explore each combination of three alternative levels of sample QC, SNP-QC and PC-correction, and two levels of CNV-QC) of the pipeline were run, varying thresholds to attempt to determine the impact on sensitivity and specificity. More detail on the pipeline development and results of testing can be found in the Supplementary Material, Methods S1–S11.

The validity of the CNV calls resulting from 54 runs of QC pre- and post-processing was assessed using several techniques, including average quality scores, comparison to a MetaboChip dataset with over 5000 samples in common with this study, comparison to the DGV and tracking bias for the overall case–control burden ratio.

This quality score was developed to filter CNVs that failed to replicate on MetaboChip. A likelihood-based score (Supplementary Material, Methods S6 for details) proved to be sensitive to the problematic artefact in the MetaboChip CNVs. Revised results filtered by quality score showed maximum sensitivity of 87.7% and specificity of 93.5%, at a cut-off of 0.95 (quality score out of 1) with an area under the curve of 0.932 (data in Supplementary Material, Fig. S5). For rDUPs, 100% of MetaboChip rDUPs were found on ImmunoChip, with 88.2% specificity.

Armed with a useful tool to evaluate the quality of calls, systematic testing varying QC thresholds revealed the most important step was PC-correction of the LRR data, particularly for rDUPs. An increasing number of components corrected-for resulted in CNV calls with substantially higher quality scores and greater correspondence with validation sets. More stringent SNP and sample QC both increased quality scores, although there was a small decrease in sensitivity suggesting that this could be at the expense of some genuine CNVs. CNV-QC steps such as excluding samples or plates with large CNV counts and excluding immunoglobin, telomeric and centromeric regions appeared to decrease the sensitivity of detection of CNVs from the DGV. However, further investigation showed many of these CNVs to be artefactual. This could suggest that some studies contributing to the DGV may have been vulnerable to the same biases, causing a spurious increase in correspondence with our dataset.

Next, running the pipeline on the family dataset using the optimal set of thresholds derived from the case–control dataset revealed several potential biases and suggested additional QC criteria. The strategy derived was to run the pipeline as above, but as a final step, to conduct further validation of any CNVR showing significant association with T1D. CNVRs with a high *de novo* rate and low transmission rate were identified as spurious CNVRs, or CNVRs that could not be detected with sufficient sensitivity across all samples. We concluded that each significant result should be vetted using likelihood-based quality scores. CNVRs that had low quality scores in a substantial proportion of samples, in almost every instance, seemed to be spurious upon manual inspection of the local LRR and BAF intensities for individual samples. CNVRs with high quality scores invariably appeared genuine based on manual inspection of the LRR and BAF data.

Finally, tracking of rates of de novo rDELs and rDUPs versus transmitted rDELs and rDUPs allowed refinement of hidden Markov model (HMM) parameters for *PennCNV.* See Supplementary Material, Methods S9 for details of this threshold experiment, which suggested slightly lowering HMM thresholds for calling DELs and duplications (DUPs). PC-correction improved the signal-to-noise ratio, pushing state means closer to zero, allowing this increase in sensitivity without loss of specificity. Relaxing the LRR threshold may have resulted in more false positives, but these seemed to be filtered effectively by downstream application of quality score thresholds, based on the consistent specificity.

*PennCNV* allows a second step of joint-calling CNVs, utilizing family relationships. The model gives a higher prior likelihood for detecting a specific CNV in a given sample when that CNV has already been found in a parent or child of that sample. Rates of transmission were pushed closer to 50% following this step, providing further confidence in the validity of the pipeline. Furthermore the success of joint calling provides a baseline to estimate the sensitivity of CNV detection when trio data are not available, such as for the case–control dataset in this study.

The remainder of this section describes the thresholds used for the final case–control burden, regional association and family trio based analyses.

### Genotype quality control (SNP-QC)

CNVs are detected using LRR and BAF scores derived from raw allele-probe intensities. In contrast to genotype calling where two SNP alleles specify three distinct genotypes, LRR combines the intensity of both probes of an allele. Genotype information, while not used directly, is a good indicator of data quality. Good call rate reflects reasonable raw intensity data, which is important for BAF and LRR values. Samples and SNPs with call rates lower than 95% were removed prior to subsequent QC and analysis steps.

Violations of Hardy-Weinberg equilibrium (HWE) assumptions can indicate poor clustering, which may also reflect poor quality intensity data. HWE cut-offs were determined by inspecting plots of HWE versus call rate to establish a threshold that was associated with low quality calls. CNPs (common CNVs) can also cause violations to HWE so if our investigations were not restricted to rare CNVs this step would be skipped.

Depending on the particular set of SNPs in a given dataset the sample distribution of mean heterozygosity should have a fairly limited range. Outlier samples in this distribution were removed as such samples are likely biased for intensity.

For GWAS, SNPs below a given minor allele frequency (MAF) threshold are typically removed. For CNV calling this step is not necessary and the SNPs can still provide LRR intensity information even when completely monomorphic in the dataset. Runs of homozygosity (ROH) are fairly common in the genome and presumably could result in reduced confidence for DEL calls as the BAF distribution for ROH may be indistinguishable from a deletion. However, based on testing of sensitivity, specificity in ROH regions there was no evidence of lower accuracy in this dataset.

SNP-QC as described above is not sufficient to prepare LRR data for CNV calling. Genomic regions with noisy LRR distributions across markers may occur even for well-defined allele-based genotype clusters as a consequence of various manufacturing and scanning processing of arrays. Additional variation can be due to laboratory-specific protocols (i.e. reagents, DNA extraction methods), all of which generate strong enough differences that generate artificial CNVs in some chromosome regions. Thus, variation in LRR values is much larger than for genotype clusters, and inference of CNV calls involves a rigorous assessment of hybridized intensity values. For example, in our study, only 14% of samples that failed LRR QC tests had a sample call rate <99%.

### Sample-QC

#### Mean exclusion

**Figure 4.**
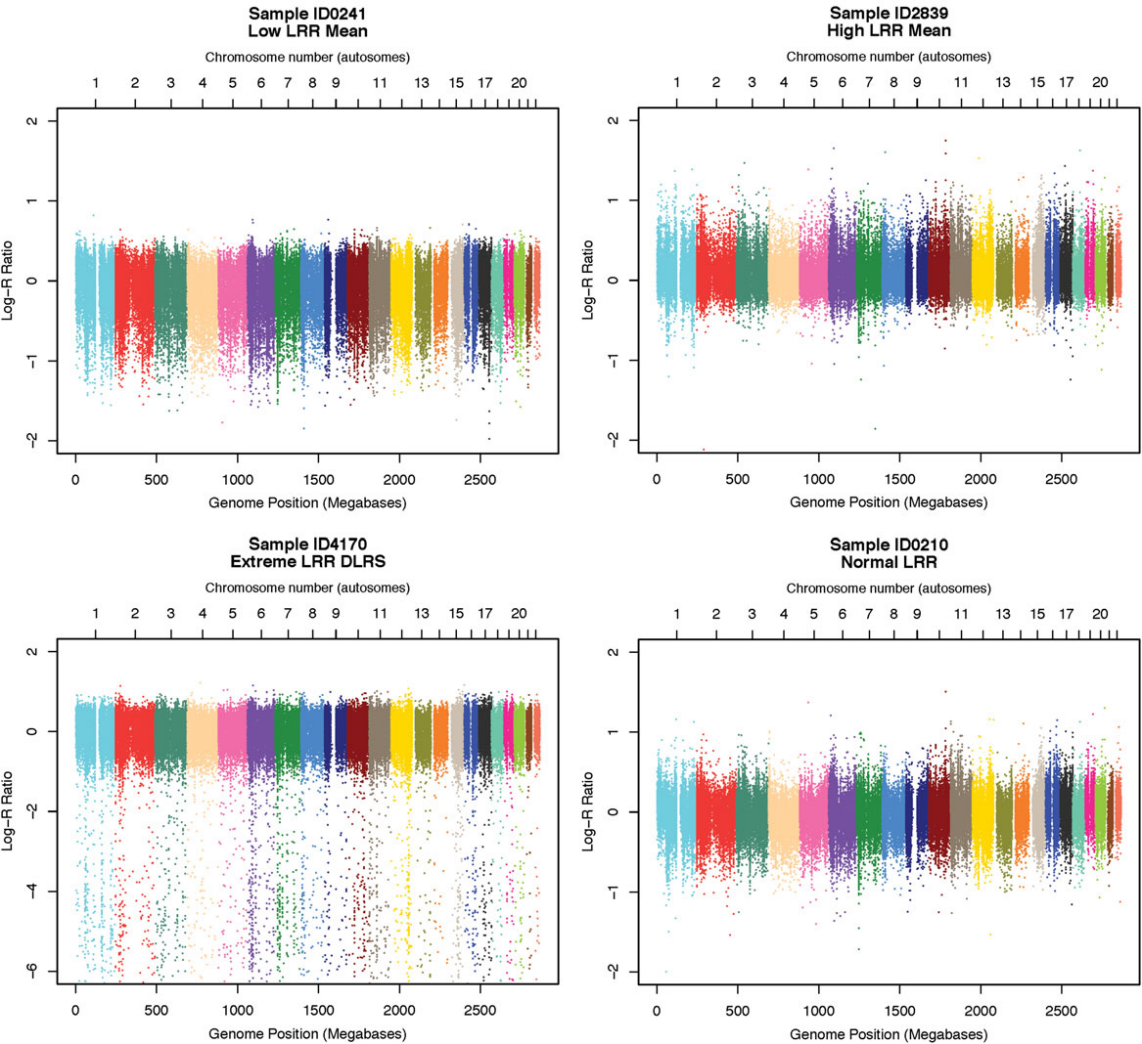
Examples of LRR artefact. Examples of sample-wise LRR data with typical types of artefact targeted during sample-QC. These are plotted across the whole genome for single samples. Each chromosome is coloured differently. From left top to right bottom: (i) low overall mean, (ii) high overall mean, (iii) high noise (DLRS) and (iv) a typical spectra (for comparison). To preserve sample anonymity the sample IDs are fake, for display purposes only, and are not connectable to any record.

Overall LRR-means were examined for each sample as extreme deviations can reflect poor hybridization, oversaturation or other cell line artefacts (Fig. 4 shows examples of high and low means). Samples were excluded that exceeded the upper or lower bound (1.5× the interquartile range) for overall mean.

#### Derivative log-ratio spread

Derivative log-ratio spread (DLRS) was calculated as the standard deviation (SD) of the differences between successive array SNP markers (ordered according to genome position), divided by the square root of 2. Noise is captured more precisely by DLRS than SD as it is more sensitive to high frequency variation, and insensitive to larger local fluctuations, such as intensity differences between chromosomes. A sample exclusion threshold was set at 3.5 SD above the cohort mean. DLRS-based sample exclusions were assessed separately in T1D cases (all genotyped at the University of Virginia, UVA), UVA-typed controls, Sanger-typed controls and the family dataset. Figure 4 shows an example of intensity data with high DLRS.

#### GC wave

Guanine and cytosine (GC) wave was evaluated to isolate samples with substantial wave intensity fluctuations. Irregular GC content, either at the probe level and/or within regions surrounding probes, is highly correlated with genomic waves in signal intensity induced by DNA quantity (69) (Supplementary Material, Fig. S6). Irregularities in GC content induce long-range wave patterns in signal intensities, an outcome known to produce high false-positive CNV calls. For samples with extreme GC-oscillations, the data were too corrupted for adjustment with the standard Diskin algorithm, so were excluded. In total, 0.9% (188 of 16 806) of samples were filtered for exceeding 3.5 SD above the mean for GC wave (Supplementary Material, Table S2). The GC score used was ‘total wave factor’ (*SWF* from (69)). It is referred to from here on as GC wave because GC variation is usually the main source of this wave artefact. Wave factor *SWF* is the median of the absolute median absolute deviation (MAD) of LRR, with sign determined by correlation with GC percentage. *SWF* was chosen ahead of the pure GC wave factor *SGCWF* because other kinds of genomic waves may impact on the LRR, affecting CNV detection.

#### Chromosome aberrations

Chromosome aberrations in the context of this study were considered to be duplications or deletions of whole chromosomes (or large parts of a chromosome). It is rare for humans to survive to birth with *aneuploidy*, but some would be expected in a large dataset. If not removed these could have a large effect on data cleaning, batch effects correction and subsequent CNV calling.

Not all of the aberrations identified using our algorithm were genuine instances of trisomies (Supplementary Material, Fig. S7) or monosomies. The remainder may reflect mosaicism, GC artefact or some samples may have had large deletions or duplications affecting a sizeable enough fraction of the chromosome to perturb the means for the whole chromosome. Regardless of whether these were genuine abnormalities, samples detected exceeding these statistical thresholds were excluded because such large blocks of extreme LRR will cause problems for PC correction and CNV detection.

#### Plate exclusion

Sample-based LRR differences between cases and controls were also examined across plates, as plate effects induced some of the strongest bias in CNV estimates, that is, they could not be validated by qPCR assays. Any plate that has a very high sample failure rate may have intrinsically poor data quality and it can be best to exclude all samples from that plate when sample size is sufficient. On average, the sample failure per plate was 11.4%, and nine samples per plate were determined to contain DELs. The majority of ‘suspect’ CNV calls for these three regions were localized to specific DNA plates. Thus, a global approach was taken that excluded plates exceeding 40% of comprising samples failing QC or plates where the number of CNVs per plate was greater than three SD above the global mean. The number of samples excluded in each dataset is described in Supplementary Material, Table S2.

#### Principal components analysis and batch correction (PC-correction)

**Figure 5.**
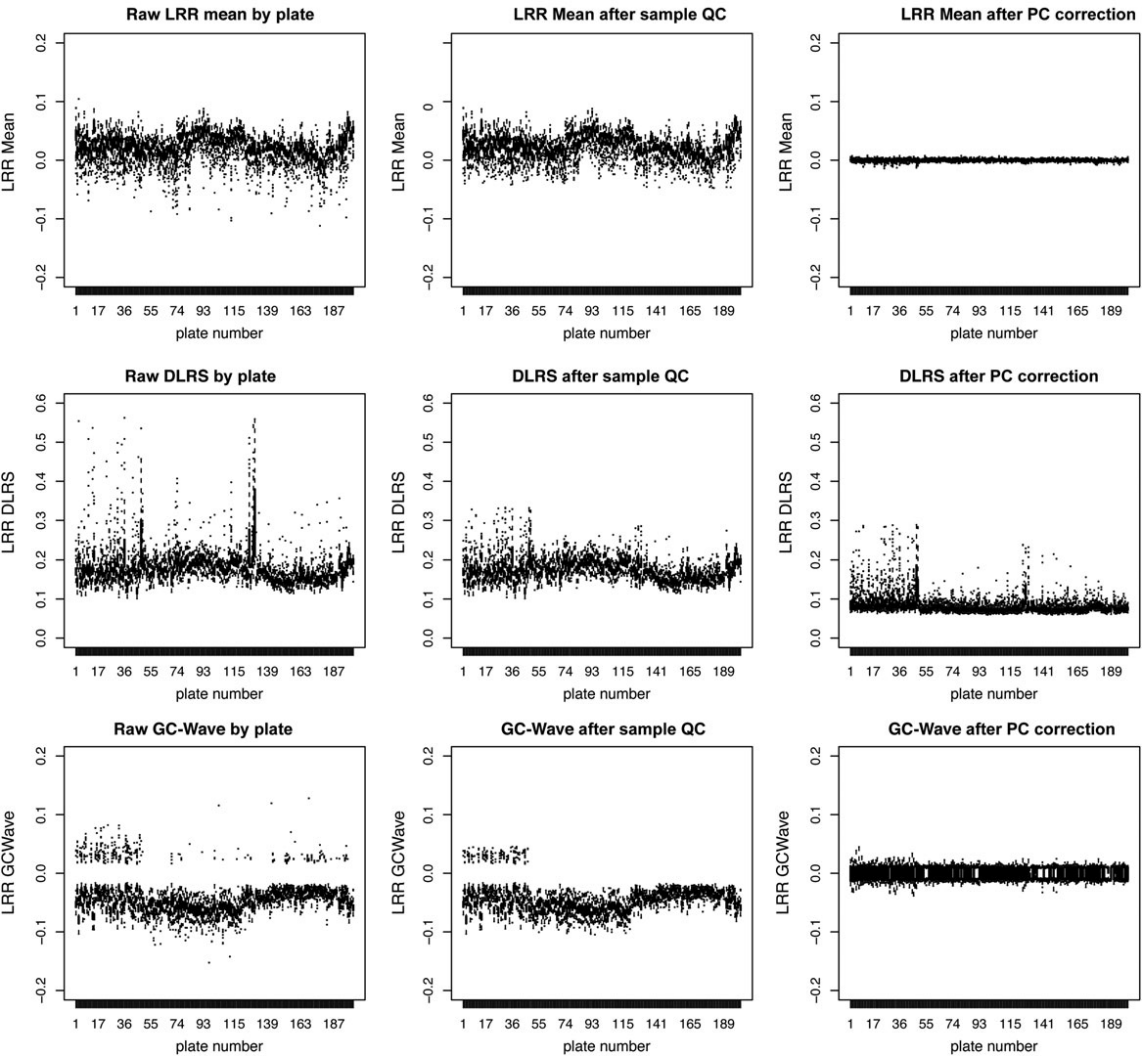
Treatment of batch effects with PC-correction. The effect on intensity distributions of applying Sample-QC (middle column), plus PC-correction (right column) to raw LRR intensity data (left column). Each row of the 3 × 3 matrix shows a different QC metric, (1) LRR-mean, (2) LRR-DLRS and (3) GC-Wave. The plots are actually boxplots across the 201 plates from the study (from each of the three cohorts, which are arranged sequentially, Sanger controls, T1D, then UVA controls). Due to the large number of boxplots on each graph they are individually indistinguishable, but the broader trends of data ‘outer’ boundaries and outlier observations (dots) are sufficient to discern the effects of interest.

Batch effects are pervasive for microarray data and LRR data are very sensitive to such. Principal components analysis (PCA) was used to correct batch effects by removing the 24 largest linear components with regression (Supplementary Material, Fig. S8). Prior to calculating PCs, SNP and samples failing QC were excluded, immunoglobin, telomeric, centromeric regions and the MHC were excluded, and SNPs were thinned to an ∼15% subset to prevent the removal of genuine CNV signals. Note that PC-correction will likely result in poor and biased detection of CNPs, as the common signals could be strong enough to influence PCs, because for CNPs a significant portion of the samples will always have a correlated intensity difference. Examination of data before and after PC-correction showed extremely successful elimination of biases and batch effects between plates and cohorts (Fig. 5).

#### Other QC criteria

Further criteria based on sex mismatch, duplicate samples or non-European/non-white ethnic group ancestry excluded 1.4% (233 of 16 806) of samples (Supplementary Material, Table S2).

### CNV calling using *PennCNV*

CNV calling was based on PC-corrected LRR for samples and SNPs passing QC. *PennCNV* was used *without* adjustment for GC-content, as the PC correction all but eliminated GC-wave (Fig. 5 and Supplementary Material, Table S1). Simulations showed that when GC wave was not present that the Diskin GC-correction implemented in PennCNV actually over-corrected and reduced the accuracy of CNV calls.

The ImmunoChip-specific population frequency of the minor allele (BAF for each SNP for calibration; distinct from the BAF signal for each SNP and sample used for CNV detection) was generated to inform CNV calls, which is a standard input parameter for the *PennCNV* algorithm.

Within high-density marker regions, *PennCNV* tends to artificially split large CNVs into smaller segments. This is a critical issue for the ImmunoChip or other highly dense arrays, as false segmentation inflates the true number of CNVs and potentially leads to the exclusion of informative samples that are mistakenly identified as carrying an extreme number of CNV calls, rather than carrying a single large CNV in a highly dense region. Furthermore, artificial segmentation of a true CNV results in partial inclusion and/or exclusion of its split segments from a given CNVR, erroneous assessment of CNV size distribution and, for family data, erroneous estimation of *de novo* CNVs. To address this issue, adjacent CNVs separated by a gap <20% the total length of the combined CNV segments were merged into a single (large) CNV.

#### CNV QC

Samples for which the total number of CNVs, or the number of rDUPs or rDELs, exceeded three SDs above the average number of CNVs per sample were excluded from analysis. In-house PCR validation has shown that such high counts usually reflect biased or contaminated data not detected through previous QC criteria.

A CNV inferred by a greater number of SNPs can usually be called with higher confidence as the influence of any outlying observation is relatively less, and the categorical state of the local BAF distribution converges with increasing observations. A rule of thumb often used for CNV calling is to only accept calls comprised of at least 10 SNPs. However, testing on this dataset suggested that six was an optimal cut-off for sensitivity and validity (Supplementary Material, Methods S3 for details on ascertaining this threshold). By convention, a CNVR was defined as ‘rare’ if the number of overlapping DELs within the region was <1% of the study population.

### T1D association analyses

#### Analysis of burden

It is possible that CNVs may explain some of the missing heritability of T1D. Given the dense sampling of T1D-associated regions on the ImmunoChip, it is plausible that there might be an overall ‘burden’ of CNVs in cases versus controls. To test this, the total rate of CNVs in cases versus controls was compared for: all CNVs, CNVs disrupting genes, CNVs disrupting exons, as well comparing rates of CNVs in different groupings of length.

#### Large CNVs

Large CNVs have a very strong a priori likelihood to cause disease. As these are likely to be unique to each sample, no analysis was possible. Nevertheless, all CNVs larger than 3 Mb were reported because of their potential functional significance. Importantly, for the case–control data we had no way of knowing whether CNVs were de novo, however for CNVs of this size, they are very unlikely to have survived selection over many generations.

#### Analysis of case–control frequency in CNVRs

To test for CNV differences at different loci, first they must be grouped in some window, as usually the start and end of a CNV near a specific locus will be slightly different between samples. The set of overlaps can be complex for a large sample, making the definition of windows semi-arbitrary. Common practice is to create CNVRs. In PLINK software (70), the command ‘–segment-group’ is used to define sets for a CNVR. The genome is first split into regions (e.g. every 1 Mb), and every CNV that overlaps that segment is initially selected. Then the region is reduced or expanded as necessary to accommodate the actual overlapping set (if any) in that range. In this way, a single CNV may sometimes end up belonging to more than one CNVR. There must be at least one nucleotide in a region overlapped by every CNV in the set. The amount of overlap within the regions may vary. This is the same grouping logic that is used to classify CNVs as ‘rare’. Some of these boundary definitions can cause spurious associations, particularly near common CNVs. For instance, it can happen that the same CNVR can be called by Plink as three separate, but overlapping, regions with slightly different boundaries. In the case of a common CNV, two of these regions could be excluded due to frequency above 1%. However, a third may by chance capture only the edge of the region and may appear in the list of rare CNVs, potentially with a skewed case–control frequency. To minimize other potential consequences of this, we raised the rare threshold to more than 1% (e.g. 3–5%) and then excluded any common CNVs with greater than 1% frequency at the CNVR analysis stage.

To analyse the CNVR frequencies, Fisher's exact test for rare events was used. This test is robust to extremely rare counts, such as occur in this dataset: e.g. frequencies as low as 1 in 8000. It can also handle zero counts—although in this case a finite confidence interval cannot be generated, only a *P*-value. Given that the most frequent possible event given our rare cut-off was 1%, power was limited. Consideration was given to multiple testing and so resulting *P*-values were compared with a Bonferroni (*p*/*k*) threshold based on the number of CNVRs tested (‘*k*’ of roughly 500, depending on which run was used, and whether rDELs or rDUPs were tested).

The standard GWAS threshold of *P* < 5 × 10^−8^ may not be appropriate here. This is because there is a greater a priori rationale that a CNV should influence disease risk, compared with a SNP (due to the larger, and clearly functional amount of DNA involved) (71).

#### Family analysis and TDT tests

Family data are very useful for CNV analysis because the accuracy of calls is increased, as a spurious call in both a parent and child is very unlikely. It also allows identification of which CNVs are de novo, and which have been transmitted from a parent. A simple case versus control frequency comparison is not appropriate for family data due to confounds of shared ancestry. Therefore analyses must be tailored around family structure.

Transmission rates were used to test for burden by taking the ratio of affected versus unaffected transmissions from parents to children. Use of this ratio for a burden calculation is probably more robust than a case–control design because ancestry and environment are equal between siblings.

Analogously to the method used in SNP analyses, a TDT test can be performed for CNVs, separately for DELs and DUPs to test for association at specific loci. For instance, in DELs normal copy-two is analogous to homozygosity for the major allele of a SNP, a single copy-one deletion is analogous to heterozygosity and a double deletion copy-zero is analogous to homozygosity for the minor allele. The normal test compares the rate of transmission from heterozygous parents to affected offspring against non-transmission. However, in the case of CNVs, sensitivity of detection is not perfect so we must also account for the rate of transmission to unaffected offspring in order to ensure that the result is not simply due to differential sensitivity of CNV detection between datasets. We performed TDT tests for all CNVRs arising in the family dataset.

#### De novo CNVs

De novo CNVs have had not been subject to selection so have a very high likelihood of influencing disease risk. These are easily identified in offspring when data for both parents are available. Equivalently to the burden calculation using transmission rates, a comparison was made between de novo rates for affected versus unaffected offspring.

Even after all the QC described, some follow-up revealed that while the de novos all appeared to be genuine CNVs, many were not genuine new mutations. Inspection of the LRR and BAF intensity at each CNV locus with comparison to any siblings, and both parents, showed clearly in many cases that the same intensity pattern was present in a parent. This was likely due to the false-negative error rate of CNV detection, so that a substantial proportion of de novo calls were incorrect due to lack of sensitivity in detecting the same CNV in a parent. This failure was most frequent when multiple CNVs labelled as ‘de novo’ appeared at the same locus. For a more common CNV, there are more chances for PennCNV to fail to detect the CNV in the parent that transmitted it. After exclusions were made via manual checking nearly all the de novo rDELs and rDUPs remaining were singletons.

## Supplementary Material

Supplementary Material is available at *HMG* online.

## Funding

This work was funded by the JDRF (9-2011-253), the Wellcome Trust (091157) and the National Institute for Health Research (NIHR) Cambridge Biomedical Research Centre. The Cambridge Institute for Medical Research (CIMR) is in receipt of a Wellcome Trust Strategic Award (100140). This work uses resources provided by the T1DGC, a collaborative clinical study sponsored by the National Institute of Diabetes and Digestive and Kidney Diseases, National Institute of Allergy and Infectious Diseases, National Human Genome Research Institute, National Institute of Child Health and Human Development, and Juvenile Diabetes Research Foundation International (JDRF) and is supported by U01 DK-062418. T1DGC supplied samples. Funding to pay the Open Access publication charges for this article was provided by the University of Cambridge RCUK block grant for Open Access.

## Supplementary Material

Supplementary Data
