# Supplementary material for: Detection and correction of artefacts in estimation of rare copy number variants and analysis of rare deletions in type 1 diabetes
Source: Hum Mol Genet. 2014 Nov 25;24(6):1774–90. doi: 10.1093/hmg/ddu581 (PMC4381751; doi:10.1093/hmg/ddu581)
Supplement: Supplementary Data [file supp_24_6_1774__index.html]

Detection and correction of artefacts in estimation of rare copy number variants and analysis of rare deletions in type 1 diabetes — Detection and correction of artefacts in estimation of rare copy number variants and analysis of rare deletions in type 1 diabetes — Detection and correction of artefacts in estimation of rare copy number variants and analysis of rare deletions in type 1 diabetes — Supplementary Data 

# Detection and correction of artefacts in estimation of rare copy number variants and analysis of rare deletions in type 1 diabetes

## Supplementary Data

Supplementary Data

**Files in this Data Supplement:**

- Supplementary Data - Pdf file
