# Supplementary material for: Detection and correction of artefacts in estimation of rare copy number variants and analysis of rare deletions in type 1 diabetes
Source: Hum Mol Genet. 2014 Nov 25;24(6):1774–90. doi: 10.1093/hmg/ddu581 (PMC4381751; doi:10.1093/hmg/ddu581)
Supplement: Supplementary Data [file supp_ddu581_ddu581supp.pdf]

SUPPLEMENTARY METHODS

1. Use of *plumbCNV for quality control, CNV calling, association and validation*

A full description of *plumbCNV* will soon be available (58). In the interim *plumbCNV* for this paper was run using the version on github: https://github.com/nicholasjcooper/plumbCNV , stored in several raw R function files, as current on the 17th June, 2014. The package primarily runs the pipeline described in Figure 3, but also contains extra functions for association, plotting, simulation, annotation, format conversion and validation of CNVs. It is comprised of ~15,000 lines of function code and uses bioconductor libraries and objects extensively. The *plumbCNV* suite requires a Linux or Mac OS X system with R 3.0 or higher, *PennCNV*, *Plink* and *BLAS* installed, and the R-package *bigalgebra* which at the time of submission required installation using the *bigpca* package function: *big.algebra.install.help*(). Calibration files include text files: (i) specifying sample ids, (ii) SNP IDs, positions and chromosomes; three additional files listing which samples belong to: (iii) each microarray plate, (iv) gender and (v) phenotype, and finally, (vi) a specification file indicating the format and locations of the raw data files containing the raw LRR and BAF data. These raw files can be genome studio files, or matrix/'long' files containing the LRR and BAF scores. The pipeline for this study was run using the one-line command: `cnvResult <- plumbcnv(settings)` , where 'settings' is a 'list' object containing file names, thresholds, and options for all stages of the pipeline. The pipeline can make use of multicore servers and clusters (e.g, via 'qsub') to significantly improve the speed of the pipeline.

2. Simulation tests for *PennCNV*

During development of the pipeline simulations were run to produce ideal datasets. The intensities of the simulated CNVs closely followed the *PennCNV* HMM assumed state distributions. Initially, this led to discovery of a problem with the *PennCNV* GC-correction (see under heading GC Wave in 'Materials and Methods'), and showed that PC-correction was biased by common CNVs. Once these problems were accounted for, we were able to confirm that all CNVs were detected based on a 'perfect' dataset. Next, we added: (i) symmetrical noise, and (ii) noise averaged over entire plates, to this ideal baseline to see how this affected detection. It was discovered by varying the noise (standard deviation (SD) parameters), that too much (greater than 1.75 times expected SD) or too little noise (less than 25% of expected SD), resulted in failure to detect simulated CNVs. In the overly noisey case, detection of false-positive CNVs also occurred. In the overly clean case, it is possible that internal parameters calibrated using the variance of the data were set to inappropriate thresholds affecting accuracy.

3. Minimum number of sites required to confidently call a CNV

It is common practice to filter CNVs with less than ten comprising sites, as there is an association of increased confidence and reliability with an increasing number of sites. However, *PennCNV* calls CNVs with as few as three sites, and *birdsuite* (Affymetrix-based caller) will call a CNV based on a single probe. The *PennCNV* markov model is dependent on the most probable sequence of states, and due to very low priors for CNV states in the transition matrix, a CNV with fewer sites must conform more

strictly to distributional assumptions if called by the HMM. Therefore, this study considered several possible cutoffs for CNVs: 4, 6, 8, and 10 sites.

For increasing number of SNPs sensitivity increased for rDELs and rDUPs, suggesting that smaller CNVs in the DGV were relatively harder to replicate. There was no effect of number of the number of SNPs cutoff for specificty in rDELs, but for rDUPs raising the cutoff was associated with lower specificity, with roughly a 5% specificity reduction going from four to ten sites, based on the beta coefficient.

Another cutoff applied after calling is the chosen percentage to define CNVs. Reasons for excluding common CNPs have been discussed although the criterion of 1% is somewhat arbitrary. However, this cutoff does provide an increase in the validity of calls, for at least two reasons. Firstly, common artifact due to batch effects (or errant QC or normalisation) is likely to affect more than 1% of samples, so artifactual calls would likely exceed this threshold. Secondly, based on simulations, very common CNPs seem to be able to sufficiently influence PC-correction so that overcorrection can create false DUPs when there are common DELs present,
and vice-versa. The 1% threshold greatly lessens the risk of this problem occurring. As mentioned in text, it can be effective to raise this threshold to between 3%-5% when doing post-CNV-QC analysis to avoid problems with adjacent regions, then exclude CNVs exceeding 1% only at the final stage of CNVR analysis.

4. Quality control approaches applied in other CNV studies

There seems to be no consensus on what quality control procedures work best, and it may be that the best approach varies between datasets and chips. Following are some representative approaches used by other groups. The first (S1) used the intersection of three CNV calling algorithms, *QuantiSNP*, *PennCNV* and *GNOSIS*.
For each they varied the QC applied. For *PennCNV* they used the built in LogR standard deviation, BAF drift and Waviness factor cutoffs. Waviness factor as is a measure of wave artifact (predominantly GC wave), is detected and corrected automatically as part of the *PennCNV* calling software. For *QuantiSNP* they used a percentage threshold for outlier probe intensities, and filtered for extreme BAF and LogR standard deviation. For *GNOSIS* they required a quality score greater than 10. For all methods they excluded samples with a large number of large negative valued probes and a SNP call rate cutoff of 98.5%. This approach yielded a positive predictive value of 91% with PCR validation in their autism dataset. The online *PennCNV* documentation recommended several quality control procedures, including those mentioned above, alongside a suggestion to remove samples or plates with large numbers of CNVs detected. The Broad Institute developed a suite of microarray analysis tools called birdsuite (S2) that incorporates a
CNV calling procedure for rare CNVs (*birdseye*) and common CNPs (*Canary*). The reported processing time is orders of magnitude slower than *plumbCNV* with estimates of 2-5 hours per 96 samples. The software only currently supports Affymetrix SNP5/SNP6 arrays. Processing requires data to be pre-prepared and treated one plate at a time. The algorithm uses plate and chromosome level intensity distributions to normalize the data within each plate. Extreme values on noise measures are used to flag samples for exclusion, alongside use of a quality score for each CNV, LOD (likelihood of duplication/deletion) which quantifies the likelihood that probes are part of a CNV rather than the copy state of the surrounding probes.

In a study designed to examine the sensitivity of a microarray-based calling method to reproduce CNVs detected by sequencing, *birdsuite* significantly outperformed two other CNV calling algorithms. Results were: 32.8%, 61.6%, 64.8%, 93.8% for CNVs with <5, 6-10, 11-20, 20+ probes respectively. Clearly CNVs tagged by a greater number of probes are more easily detected. The other methods (Nexus, Partek) showed lower sensitivities in this test: 5.5%, 42.0%, 69.0%, 74.4% for Nexus with 'relaxed' settings, and 0.4%, 13.4%, 47.9%, 72.9% for Partek [S2]. Most LRR-QC approaches use filters based on extreme intensity values. Many apply SNP quality control prior to calling (e.g, call rates), and filter by some sort of quality score. *Birdsuite* seems to perform well based on validation metrics, however the process is slow and cumbersome (though comprehensive) and limited to Affymetrix arrays. The Sanders method, implemented as a pipeline called *CNVision* was shown to perform well in the context of several autism case control studies. However, for application to the current dataset the method in [S1] has no way to deal with systematic intensity biases between cohorts collected and typed separately.

5. Validation using common samples with a MetaboChip dataset

To test the reliability of CNV calls from this pipeline, CNV detection and QC was run on a set of 5,458 samples that had also been typed on another Illumina custom iSelect chip, MetaboChip, which has 5.9% SNPs in common with ImmunoChip (n=11,622). CNVs detected by MetaboChip were filtered to select only those covered by at least five ImmunoChip SNP positions (regardless of whether they were the same SNPs). Then the Immunochip data in these locations was tested for the presence of the same CNV in the same sample. Sensitivity for rDELs was initially low but inspection of the data showed this was due to a specific artifact in the MetaboChip data, caused by a high rate of monomorphic SNPs. This artifact was clearly identifiable on visual inspection but had not been picked up by any QC. This prompted the creation of a CNV quality score.

6. CNV Quality Score

The score was comprised of two parts: (i) a BAF quality score based on the emmisions matrix for the *PennCNV* HMM, namely the likelihood that the CNV SNP intensities were in a CNV state and that the surrounding SNP intensities reflected a normal state; (ii) an LRR quality score based on the percentage of CNV SNP intensities on the expected side of zero, versus the percentage of SNP intensities on and around zero for the surrounding region.

This quality score proved to be sensitive to the problematic artifact in the MetaboChip CNVs. Revised results filtered by quality score showed maximum sensitivity of 87.7% and specificity of 93.5%, at a cutoff of .95 (quality score out of 1.0) with an area under the curve of .932. For rDUPs, 100% of MetaboChip rDUPs were found on ImmunoChip, with 88.2% specificity, regardless of whether the quality threshold was applied. Note that the quality scores have only been shown to correct the artifact that was causing problems in the MetaboChip rDEL set, not in ImmunoChip DELs. Furthermore, the number of detected CNVs in the same samples was only 95 rDELs and 54 rDUPs, so it is possible that this correspondence between chips is not representative. See supplementary Figure 5 for a plot of quality score against number of SNPs per CNV (more tagging SNPs generally thought to increase quality).

The score is implemented in the *plumbCNV* function '*process.quality.scores*()', which can be run on any existing *plumbCNV* session, but is also automatically run during normal operation, so quality score tables should be available in the *plumbCNV* output folder 'RESULTS'. We found that a quality score threshold of 0.95 for rDELs and 0.7 for rDUPs was optimal for our dataset. Another extremely useful criteria implemented was to exclude an entire CNVR if a large proportion of CNVs in that CNVR were excluded for having a low quality score. This threshold is quite artibitrary, but for higher confidence we recommend a stricter threshold. For instance, we have widely used 80% as a threshold (80% in a CNVR must pass the quality score threshold), which we observed was effective at identifying CNVs known to be spurious that had passed other QC criteria.

7. Systematic correlations of gender and age with LRR intensity

A previous study (43) using the same T1D family dataset as the present study identified a number of technical artifacts including an age effect on intensity at chr14q11.2, chr7q34, and chr14q32.33. The current dataset was tested using genome wide correlations for the same effect but there were only three locations passing a bonferroni adjusted p-value for an age effect, and these did not show the same sort of smooth effect when plotted against age, and occurred in unconnected regions. Targeted plotting of SNPs tagging the same regions did reveal a similar shaped trend at some sites to those in Figure 3 of (43), however these did not reach statistical significance at an adjusted p-value, and furthermore the majority of locations in these 3 regions showed inconsistent shapes. So it was concluded that these location specific age intensity confounds seen previously (43) were not observed convincingly in the current dataset.

Gender differences in intensity are a commonly observed phenomenon, particularly in the context of genotype calling where males and females can have slightly offset cluster centres. Genome wide associations between LRR intensity and gender were significant at an adjusted p-value at 32.1% of all SNP locations prior to PC-correction. After 24-PC correction 8.2% sites still showed differences, although *plumbCNV* has an option to correct for gender differences, which, as expected, left no differences.

8. PCR validation of biases

The most notable plate-specific artefacts (Methods) were detected for three gene regions:
(1) within the *HOXA1-HOXA13* set of paralogous genes at chromosome 7p15.2 for a 46.5 kb CNVR, with 30:2 case:control rDEL calls,
(2) 20 kb downstream from the common HLA CNVR at 6p21.33 for a 1.2 kb CNVR that included 23:1 case:control rDEL calls at *HCG9*, and
(3) on 12q14.1 at *AGAP2* for an 11 kb CNVR with 9:0 case:control rDEL calls.
One of the 15 excluded plates consisting of the highest number of rDEL carriers (n=37) had a sample-based LRR mean of 0.02 and DLRS of 0.09, well within the normal limits detected across all other plates (see Figure 9 for plate-based LRR and DLRS distributions). Thus, the artefactual CNV calls on this plate were not identified through the array-wide LRR and DLRS metrics.

Given the repetitive architecture of the 13 *HOXA* genes, we tested the *HOXA1*-*HOXA13* region for duplicate mappings of ImmunoChip assays and tested the identity between the paralogous genes. No evidence for cross-hybridisation for any of the probes was detected, and none of the overlapping genes contained sequence duplication. The *HCG9* rDEL calls, in immediate vicinity (20 kb) of a common CNVR in the HLA region, may reflect a boundary-specific spurious signal or, as referred by other recent studies, a 'peninsula' effect of a common CNVR generated by variation in boundary truncation in CNV calling. None of the *HOXA1-HOXA13* rDELs were successfully validated with qPCR, using ABI Hs02286016_cn, Hs00252571_cn, Hs03653822_cn, and Hs03623434_cn assays. Since no qPCR assays were available for the *HCG9* rDEL, a conventional PCR and agarose gel analysis was used with two sets of primers: A_F accgcgtaccaaacaataatataga, A_R cttcctattgctgtggggactat, and B_F aattcccaataaccaaattcatagag, B_R ctaactttctggtgctgcctttt. We could not validate the *HCG9* rDEL.

For the third plate-specific CNVR, we were able to validate the nine *AGAP2* rDEL calls for the T1D cases by using ABI qPCR assays Hs07012691_cn and Hs02280355_cn. However, since we did not observe any rDEL calls in controls, and cell line production and propagation can be associated with genomic mutations and CNVs, we analysed an additional 96 cases and 96 controls using blood (genomic) DNA to confirm the frequency of *AGAP2*. qPCR assay of these blood DNA samples showed high variability in copy number, with the largest variation caused by DNA extraction method (chloroform versus Qiagen kit). In addition, we also found high variability in CNVR copy number within each of the DNA preparation methods. This difference was present across multiple probes in the *AGAP2* CNVR (Supplementary Figure 16). The probe sets, although validated by the manufacturer, did not perform reproducibly in DNA samples isolated by different methods. It may, therefore, be possible that impurities in DNA samples may contribute to unpredictable changes and discrepancies in this region when analysed by array or PCR assays, as we have proposed previously (S3). Therefore, given the plate-specific artefacts, all samples from the outlier plates were removed, removing the enrichment initially detected for these three CNVRs.

List of PCR replication locii

The following loci were subjected to validation using PCR during the validation of the CNV-QC pipeline. Those that failed to replicate were generally CNVs that showed a case-control association before the QC method was refined to deal with a new type of artifact.

(continued over page...)

Failed to replicate:
ADAMTS7, AGAP2, AIRE, BCAR1, C20orf94, CABP7, CTRB2, DEAF1, DGKQ, FOXP3, HCG9, HOXA3, IFIH1, INS, PTPN2, SLC26A1, STARD3, TCAP, TH

#	Chr	Start	End	gene	band
1	2	162,831,836	162,883,285	*IFIH1*	q24.2
2	4	942,676	957,344	*DGKQ*	p16.3
3	4	962,865	977,224	*SLC26A1*	p16.3
4	6	30,050,848	30,054,175	*HCG9*	p21.33
5	7	27,112,334	27,158,458	*HOXA3*	p15.2
6	11	634,225	685,740	*DEAF1*	p15.5
7	11	2,124,432	2,139,027	*INS*	p15.5
8	11	2,131,627	2,149,611	*TH*	p15.5
9	12	56,405,251	56,422,209	*AGAP2*	q14.1
10	15	76,838,601	76,890,828	*ADAMTS7*	q25.1
11	16	73,795,496	73,798,573	*CTRB2*	q23.1
12	16	73,820,429	73,859,452	*BCAR1*	q23.1
13	17	35,046,938	35,073,248	*STARD3*	q12
14	17	35,073,966	35,076,326	*TCAP*	q12
15	18	12,775,485	12,874,334	*PTPN2*	p11.21
16	20	10,363,949	10,565,012	*C20orf94*	p12.2
17	21	44,530,191	44,542,530	*AIRE*	q22.3
18	22	28,446,344	28,457,818	*CABP7*	q12.2
19	X	48,994,354	49,008,232	*FOXP3*	p11.23

*Supplementary Methods Table 1**:** Summary of rDELS failing replication using PCR.*

Successful replication:

DAP3, GLIS3, KIR3DL2, MKKS, PKIA, SH2B1*:

#	Chr	Start	End	Gene	Band
1	1	153,925,473	153,975,425	*DAP3**	q22
2	8	79,590,891	79,678,040	*PKIA*	q21.12
3	9	3,814,128	4,290,035	*GLIS3*	p24.2
4	16	28,782,725	28,793,027	*SH2B1*	p11.2
5	19	60,053,710	60,070,474	*KIR3DL2*	q13.42
6	20	10,333,839	10,362,866	*MKKS*	p12.2

*Supplementary Methods Table 2**:** Summary of rDELS replicated using PCR.*

*Note that *DAP3* was only replicated in African Americans. *KIR3DL2* CNVs were successfully detected in this analysis, but excluded as the incidence was above the 1% threshold for rare CNVs.

Successful qPCR validation of two CNVRs

During QC of CNVRs, it became apparent that quite often the CNVRs with the most significant case-control p-values, were the most affected by artifact. After refining the QC to a level that seemed to produce valid results, we tested some of the top hits using qPCR. Even though with bonferroni correction these were not confirmed case-control differences, these are still probably the best targets to demonstrate that the pipeline can produce valid CNV calls that can differ between cases and controls.

rDELs at *PKIA*, a gene located on chromosome 8q21.12 had nominal evidence for association with T1D, with six case rDELs and one control rDEL of median size 23 kb (OR=8.59, p_{FET} = 0.022). *PKIA,* is a member of the cAMP-dependent protein kinase (PKA) inhibitor family, and is a potent competitive inhibitor of cAMP-dependent protein kinase activity. All *PKIA* rDELs were confirmed with quantitative polymerase chain reaction (qPCR) (Hs06173614_cn, Hs06242849_cn and Hs05059220_cn, Supplementary Table 7).

Another large CNVR, ~500 kb on chromosome 16p11.2, has been widely reported in the literature as contributing to the genetic risk of obesity (11–15). All *SH2B1* CNVs tested (rDELs and rDUPs) were confirmed by qPCR (Hs02927996_cn and Hs01749891_cn, Supplementary Table 7). To explore whether there was any evidence for an effect of copy number dosage for *SH2B1* rDUPs, we also compared *rDEL* frequencies amongst the case-control and family datasets (Supplementary Methods Table 3). Each of these were replicated using PCR.

A. Case-control data

Locus	*Start (Mb)*	*End (Mb)*	*Length (kb)*	Genes	Cases	Controls	FET (p)
16p11.2	28.39	28.93	540.1	*SH2B1 + 14*	7	3	0.1030

B. Family data

Locus	*Start (Mb)*	*End (Mb)*	*Length (kb)*	Genes	Cases	Controls	FET (p)
16p11.2	28.73	28.93	203.2	*SH2B1 + 14*	6	0	0.0210

Supplementary Methods Table 3: Summary of rDEL counts at the 16p11.2 locus for the case-control (A) and family (B) datasets respectively. 'Case and control' labels are used in the family counts instead of 'affected and unaffected' because these counts include four parents. Construction of this table was done 'post-hoc', prompted by a nominal result in the rDUPs summary table for SH2B1. Follow up in rDELs revealed an effect consistent with the rDUPs result, where increasing copy number, from one to two to three, is associated with control status. In the 'Genes' column, '+n' indicates that in addition to the gene indicated a further 'n' genes overlap that CNV. Coordinates are UCSC hg18.

Testing the overall affected versus unaffected ratio in the family data using FET is not recommended due to irreconcilable confounds of family structure. However, for this rDEL, there was only one trio in which a parent transmitted to an affected child (two were de novos and the remainder were found only in parents) so the TDT analysis had no chance to capture an effect. Combining the results for the family and case-control datasets resulted in 13 rDELs in T1D samples to three in controls. N

9. Assessment of Sensitivity and Specificity when varying HMM parameters

Tests were run on the family dataset to calibrate the sensitivity of duplicate and deletion HMM parameters, by examining denovo and transmission rates. Testing suggested that the expected value for detecting copy 'three' DUPs should be slightly lowered from the default, which may better deal with the intrinsically lower effect size of LRR change for duplications. Using the copy-three rDUP expected value of 0.2 instead of 0.4, increased sensitivity by ~15% whilst maintaining specificity. The expected value for detecting copy 'one' DELs was changed from -0.65 to -0.55, based on small improvements in de novo and transmission differences with and without

trios, alongside a 4% improvement in specificity and more than 15% improvement in sensitivity. The stronger threshold was avoided due to the increased de novo rate. Note that the de novo rates with trio calling are higher than reported in the main paper (e.g, ~6% versus ~1%), this is because these estimates include CNVs with population frequency between 1% and 5%, and because exclusions from manual checking of intensity plots were not applied.

rDELs

	HMM	Trans-mission	De novo	CNV count	Specificity	Internal Sensitivity	Max-set Sensitivity
**COPY 1 MEAN= -0.65***	replicate 1	41.2%	14.9%	1655	60.5%	86.2%	65.7%
	with trio-calling	46.7%	6.1%	1162			
**COPY 1 MEAN= -0.65***	replicate 2	41.5%	13.2%	1561	63.9%	84.3%	65.3%
	with trio-calling	45.7%	6.4%	1182			
**COPY 1 MEAN= -0.55**	RDEL	39.9%	14.8%	1951	64.4%	87.6%	82.3%
	with trio-calling	44.1%	6.1%	1434			
**COPY 1 MEAN= -0.40**	RDEL+	41.1%	19.6%	1932	65.8%	83.4%	83.4%
	with trio-calling	43.4%	9.5%	1526			

rDUPs

	HMM	Trans-mission	De novo	CNV count	Specificity	Internal Sensitivity	Max-set Sensitivity
**COPY 3 MEAN = 0.4***	replicate 1	36.3%	30.5%	2239	66.1%	88.0%	72.5%
	with trio-calling	45.1%	16.0%	1683			
**COPY 3 MEAN = 0.4***	replicate 2	36.4%	30.6%	2262	65.6%	88.3%	72.6%
	with trio-calling	44.7%	16.9%	1680			
**COPY 3 MEAN = 0.3**	RDUP	37.5%	27.7%	2536	65.3%	81.5%	81.0%
	with trio-calling	44.8%	15.9%	2031			
**COPY 3 MEAN = 0.2**	RDUP+	37.3%	27.9%	2701	66.5%	87.9%	87.9%
	with trio-calling	43.1%	17.2%	2043			

Supplementary Methods Table 4: Comparison of CNV calling sensitivity and specificity for differing HMM parameter values. The first column shows the variations on the LRR mean parameter for rDEL and rDUP state respectively. Results are presented for with and without joint trio calling, to show the improvement in accuracy for each. Specificity is calculated by looking at the number from the non-trio set not found in the cleaner trio set. Sensitivity is the proportion of final trio called sets that were present in the non-trio sets. Max-set sensitivity is relative to the sets from the most sensitive HMM setting.

Each pair of rows of Supplementary Methods Table 4 compare CNV calling performance without, then with trio-calling. Trio calling generally results in less CNVs, as whilst some extra CNVs are identified, more are discarded due to not being present in other family members, and the confidence of the call being too low to claim a de novo. Transmission is the percentage of CNVs in parents passed to all children.

The de novo estimate is the number of CNVs found in children but not in either of their parents. The CNV count is the final number of rDELs or rDUPs in that particular test run. The specificity percentage is the rate of CNVs in the set that doesn't use trio-calling, versus the set that does. The internal specificity is the percentage of (validated within a family member) trio-called CNVs that were present in the original set without trio-calling. The max-set specificity uses the run with the highest sensitivity to set the denominator for the sensitivity percentage, instead of the current run. A copy-1 (rDEL) mean of 0.55 was judged to be optimal based on specificity and sensitivity comparable to the mean=0.4 run, but with lower de novo rates. A copy-3 (rDUP) mean of 0.2 was judged to be optimal based on maximising sensitivity and specificity whilst showing de novo and transmission rates comparable to the runs using more conservative thresholds. Note that specificity is stable alongside increases in sensitivity, this is due to the moderating influence of quality scores, which filter out many false positive hits. *= default PennCNV parameters, HMM model means: Copy 1 ~ -0.65, Copy 3 ~0.4.

10. Estimating CNV detection accuracy via de novo and transmission rates

An estimate of the overall sensitivity of the CNV detection pipeline, incorporating the limits of the technology, datasets, and the impact of the QC, can be made using the transmission rates in familial samples (for which the calling was not informed by the family structure) divided by the expected transmission rate of 50%. Trio calling using family data uses extra prior information (family structure) in addition to the LRR and BAF signals to call CNVs. For instance, if a CNV is found in a child but not a parent, or vice versa, that region may be reevaluated within that family for a CNV using a more relaxed detection threshold. Trio calling should yield rates of transmission closer to the expected 50% with a correspondingly lower frequency of de novo CNVs. This estimate (derived by doubling the overall percentages in the top two rows of Supplementary Methods Table 5) was 79.8% for rare CNV deletions (rDELs) and 74.6% for rare CNV duplications (rDUPs). The estimate represents a lower bound because some of the CNVs *not* transmitted are likely to be false positives. An upper bound was estimated as the proportion of final trio called sets of rDELs and rDUPs that were present in the non-trio sets, yielding 87.6% for rDELS and 87.9% for rDUPs. An upper estimate of the specificity was equivalently made using the change in *de novo* rate with trio calling, as the 'de novos' that are excluded once family data is better accounted for can confidently be called false positives. 91.3% for rDELs [1 - (14.8 - 6.1%)], and 89.3% for rDUPs [1 - (27.9-17.2%)]. A lower bound estimate for the specificity was derived from the percentage of the non-trio sets not found in the trio called sets. This lower bound was 64.4% for rDELs and 66.5% for rDUPs.

So, the lower and upper estimates produced were:

**rDELS:**	**rDUPs:**
Sensitivity: 79.8% - 87.6%	Sensitivity: 74.6% - 87.9%
Specificity: 64.4% - 91.4%	Specificity: 66.5% - 89.3%

These results are relatively consistent with an overall Mendelian inconsistency rate of ~20% found for *PennCNV* in an independent study (46). See Supplementary Methods Table 5.

Family data without trio calling

De novo	*Affected*	*Unaffected*	**Transmission**	*Affected*	*Unaffected*	*Overall*
rDELs	15.2%	13.3%	DELs	39.9%	39.9%	39.9%
rDUPs	27.1%	29.9%	DUPs	35.3%	40.2%	37.3%

Family data with trio calling

De novo	*Affected*	*Unaffected*	**Transmission**	*Affected*	*Unaffected*	*Overall*
rDELs	6.6%	3.6%	DELs	44.2%	43.8%	44.1%
rDUPs	17.4%	16.7%	DUPs	42.3%	46.4%	43.1%

Supplementary Methods Table 5: Analysis of de novo and transmission rates for CNVs within family trios, in affected and unaffected, with and without using joint 'trio-calling' For all transmission percentages the denominator is the total number of possible transmissions for that category (i.e., the number of children multiplied by number of CNV-carrying parents in each family for each CNV, summed across all CNVs). For the de novo percentages, the denominator is the total number of CNVs found in children with at least one parent in the dataset. Additional data for de novo and transmission rates can be found in Supplementary Methods 9. Note that there are roughly four times more affected than unaffected children in the family dataset. Prior to generating these percentages, CNVs were excluded from CNVRs that did not have at least one CNV with a quality score above 90%.

Furthermore, for a more direct comparison with the larger case-control dataset, this transmission ratio was alternatively calculated without using the family structural information for CNV calling. The rates for rDELs were still equal, giving a ratio of 1.0 (39.9% for both affected and unaffected), showing that there was no bias, regardless of whether the greater accuracy afforded by joint calling was available.

11. Control versus control testing

For overall CNV counts versus samples passing QC, the ratio between the two control cohorts was 0.99 (Sanger: 573/4213, UVA: 691/5025) for rDELs and 1.05 (Sanger: 1151/4213, UVA: 1306/5025) for rDUPs.. These cohorts were genotyped at different times in different centres, and had different proportions of samples with cell-line versus genomic DNA samples, which were major potential sources of bias that seem to have been overcome.

In order to further demonstrate lack of bias for burden tests, a comparison over lengths was made between the UVA and Sanger control cohorts. It shows that despite batch effects, there was still a stable ~1.0 ratio between control groups (Supplementary Methods Table 6).

A. rDELs

length	Sanger	UVA	Odds-Ratio	95% C.I.	FET-p-value
0 – 20 kb	309	411	0.898	0.768,1.049	0.174
20 kb – 400kb	235	249	1.143	0.948,1.379	0.16
> 400 kb	29	31	1.127	0.654,1.936	0.697

B. rDUPs

length	Sanger	UVA	Odds-Ratio	95% C.I.	FET-p-value
0 – 50 kb	453	529	1.034	0.904,1.183	0.637
50 kb – 400kb	576	644	1.088	0.963,1.229	0.177
> 400 kb	122	133	1.107	0.856,1.431	0.444

Supplementary Methods Table 6: An analysis of control versus control burden for rDELs (A) and rDUPs (B) across three subsets of CNV length. Each row contains results where the CNV set is filtered to include CNVs within the size window indicated ('Length'). There is no burden for this length-subset analysis on the UVA (control) versus Sanger (control) groups. Locations used hg18 coordinates.

12. Examing the distribution of CNV lengths between cases and controls

Differences in length distribution could either indicate systematic differences in array sensitivity or a widespread burden effect. The length distribution of CNVs was examined in cases versus controls. Conventional GWAS arrays detect CNVs at the minimum 20 kb threshold with a high degree of confidence. In contrast, the ImmunoChip allowed reliable identification of CNVs much smaller than 20 kb that were detected in 62% of rDEL carriers. Overall, we did not find a significant difference in rDEL or rDUP length between case and control carriers (P>.05), with nearly identical medians, and first and third quartiles. For rDELs, a median size of ~13 kb was estimated in both cases and controls, ranging between 0.6 kb and 23.5 Mb, and with first and third quartiles of 6.7 kb and 33.5 kb. rDUPs were substantially longer, with a median size of ~86 kb in both cases and controls, ranging between 0.6 kb and 9.7 Mb, and with first and third quartiles of 28.1 kb and 196.7 kb.

The largest rDEL in the case-control dataset was 23 Mb located on chromosome 6 (position 97.55-121.05 Mb). This result could have been biased by the chromosome aberrations QC, which excludes samples that have high or low intensity for an entire chromosome. The algorithm is designed to automatically exclude samples with aneuploidy, but could also be triggered by very large CNVs. So feasibly, a higher rate of sample exclusion for controls with very large CNVs could have influenced this burden result. However, the opposite was true. The relative frequencies of outlier samples excluded for extreme chromosome mean intensities were consistent with the burden result, whereby T1D cases had a higher frequency of chromosome-wide intensity differences than controls, OR=1.51, FET(175,183), p-value = 1.4×10^{-4}.

Supplementary References:

S1) Sanders, S. et al. *Multiple Recurrent De Novo CNVs, Including Duplications of the 7q11.23 Williams Syndrome Region, Are Strongly Associated with Autism*. Neuron 2011, 70, 863–885.

S2) Korn, J. M. et al. *Integrated genotype calling and association analysis of SNPs, common copy number polymorphisms and rare CNVs*. Nature genetics 2008, 40, 1253–1260, PMID: 18776909.

S3) Field, S.F., et al., *Experimental aspects of copy number variant assays at CCL3L1*. Nat Med, 2009. 15(10): p. 1115-7.

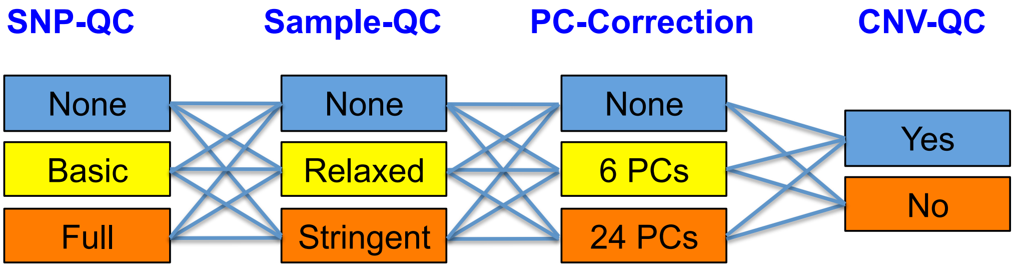

Supplementary Figure 1: *Schematic for QC iterative calibration*
Schematic of the 54 runs of the QC pipeline used to test the quality and sensitivity of results given by various thresholds. The 54 runs were formed by three by three by three by two combinations of applied thresholds (3 x 3 x 3 x 2 = 54).

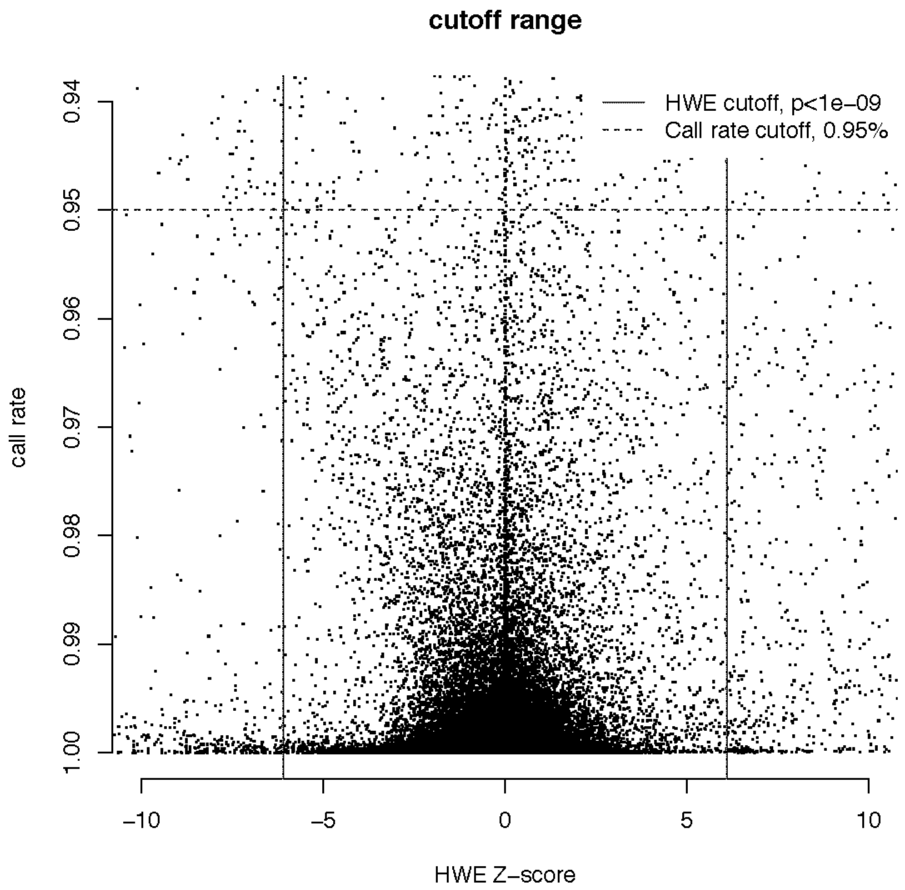

Supplementary Figure 2: *HWE versus SNP call-rate*
HWE Z-score versus SNP Call rate for all SNPs on immunochip. Plotting these two metrics together helps with choosing outlier cutoffs that preserve SNPs that do not violate a normal distribution.

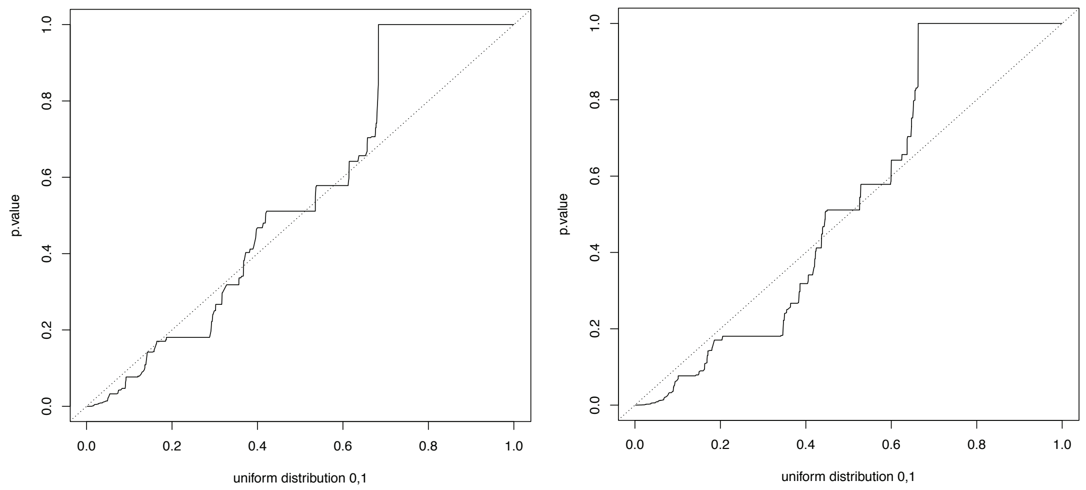

Supplementary Figure 3: *Quantile-quantile plots for FET p-values*
QQ plots for rDEL (left) and rDUP (right) p-values from FET analyses of CNVRs. The observed values closely follow the theoretical values showing the analyses was essentially unbiased, without inflation. The stepped shape of the line is due to the discrete nature of the data, so only specific p-values are observed.

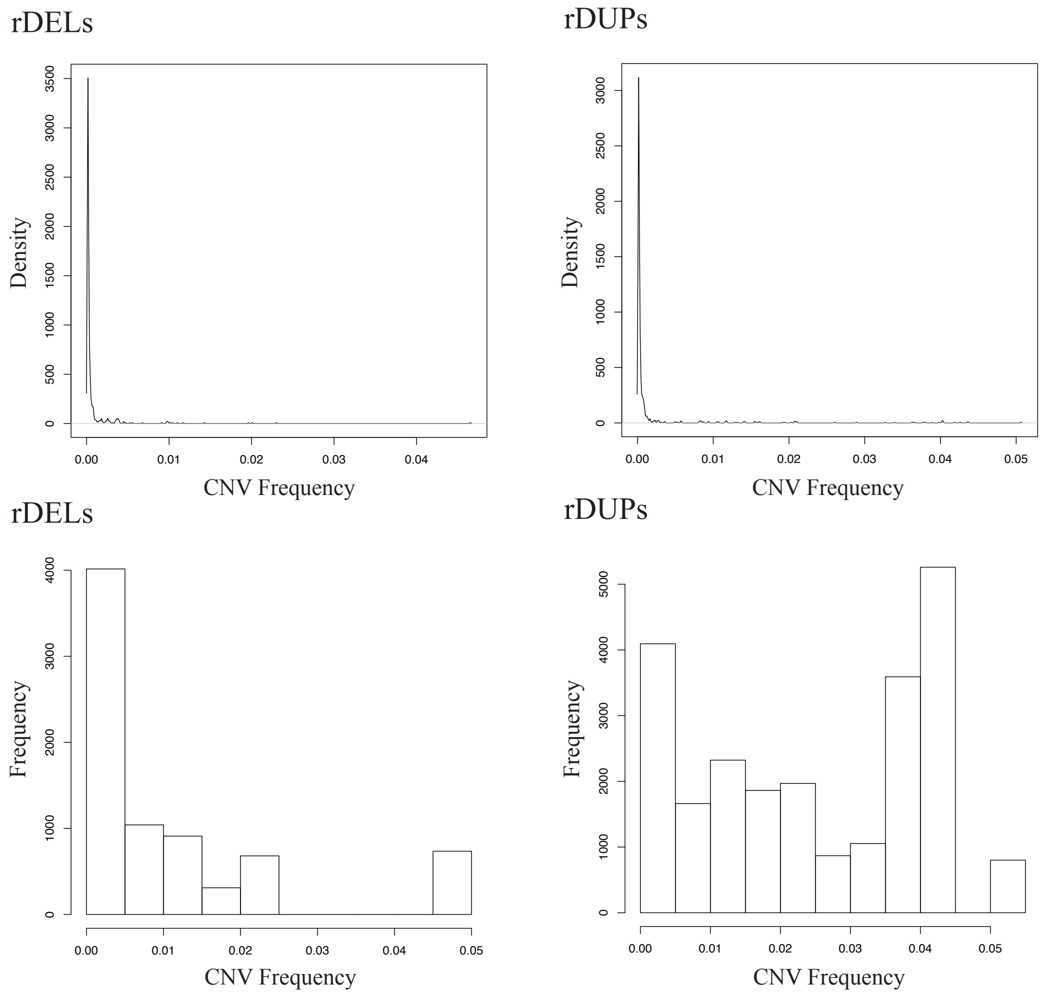

Supplementary Figure 4: *Frequency distributions of CNVs across regions and samples*

Frequency for rDELs (left) and rDUPs (right) when CNVRs > 5% are filtered The top two plots show frequency by region, showing that most unique CNVRs are rare, whereas the bottom two show frequency by sample, showing that because there are more instance of more common CNVs/CNPs, the overall distributions become more uniform across frequency.

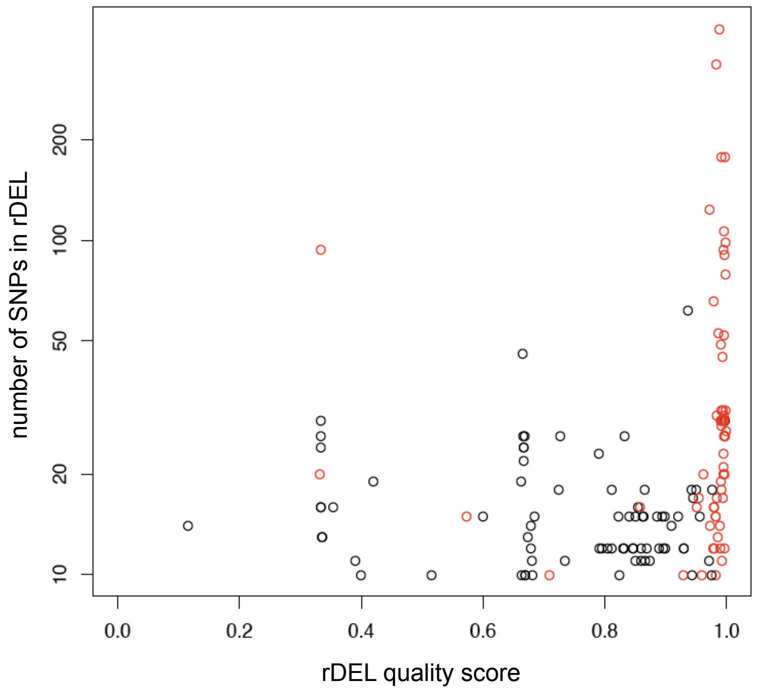

Supplementary Figure 5: *Number of SNPs per rDEL, versus quality score*
Plot of the number of SNPs in Metabochip rDELs for the 1958BC (UVA group for ImmunoChip data) samples versus quality score. rDELs validated by inspection are coloured red. It can be seen that a high quality-score cutoff captures most of the validated (red) datapoints.

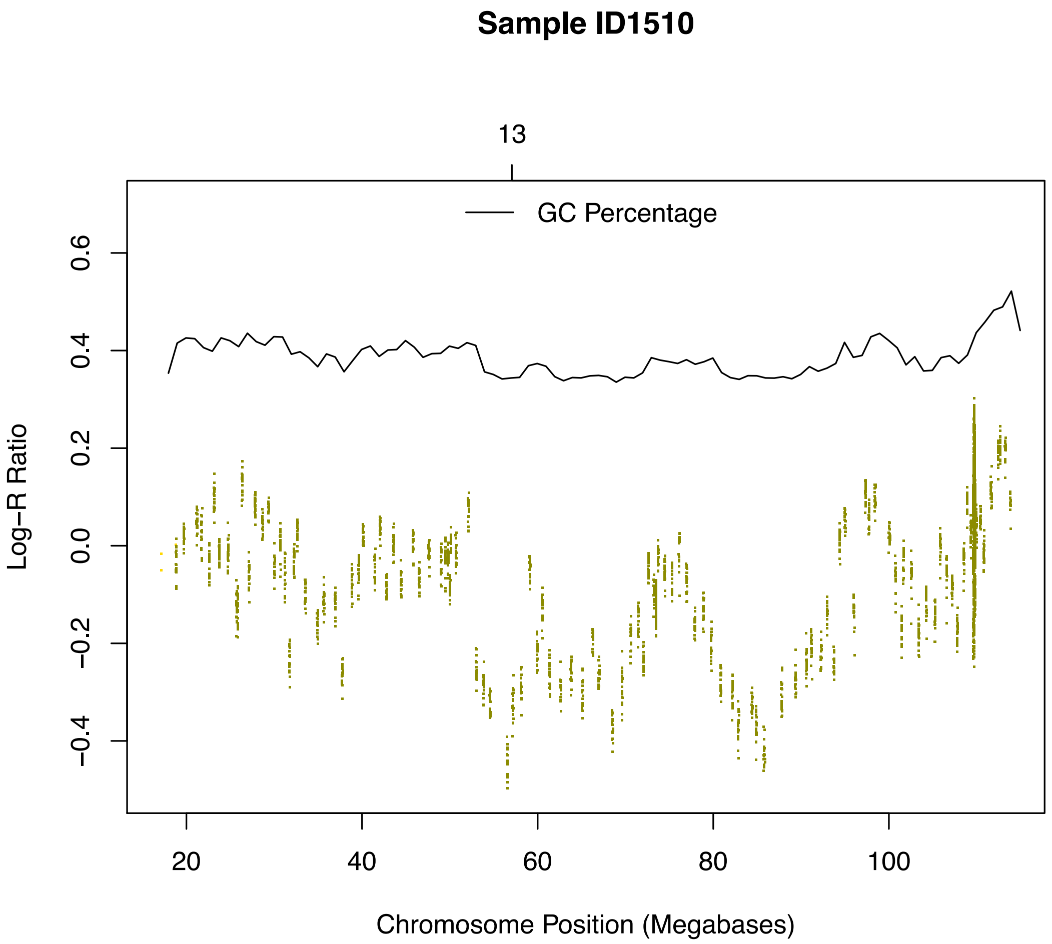

Supplementary Figure 6: *Exemplar data for GC wave artifact in a single sample*
An example of GC Wave, showing a close look at the LRR intensity for a single sample, overlaid with GC. The LRR (green wave) is median-smoothed at a resolution suited to highlight the wave fluctuations, which results in a narrower y-axis range than the previous LRR plots. The black line above the LRR observations is GC percentage, calculated for each 1 megabase window. Each significant GC peak corresponds with a major LRR peak. To preserve sample anonymity the sample ID is a fake id for display purposes only and is not connectable to any record.

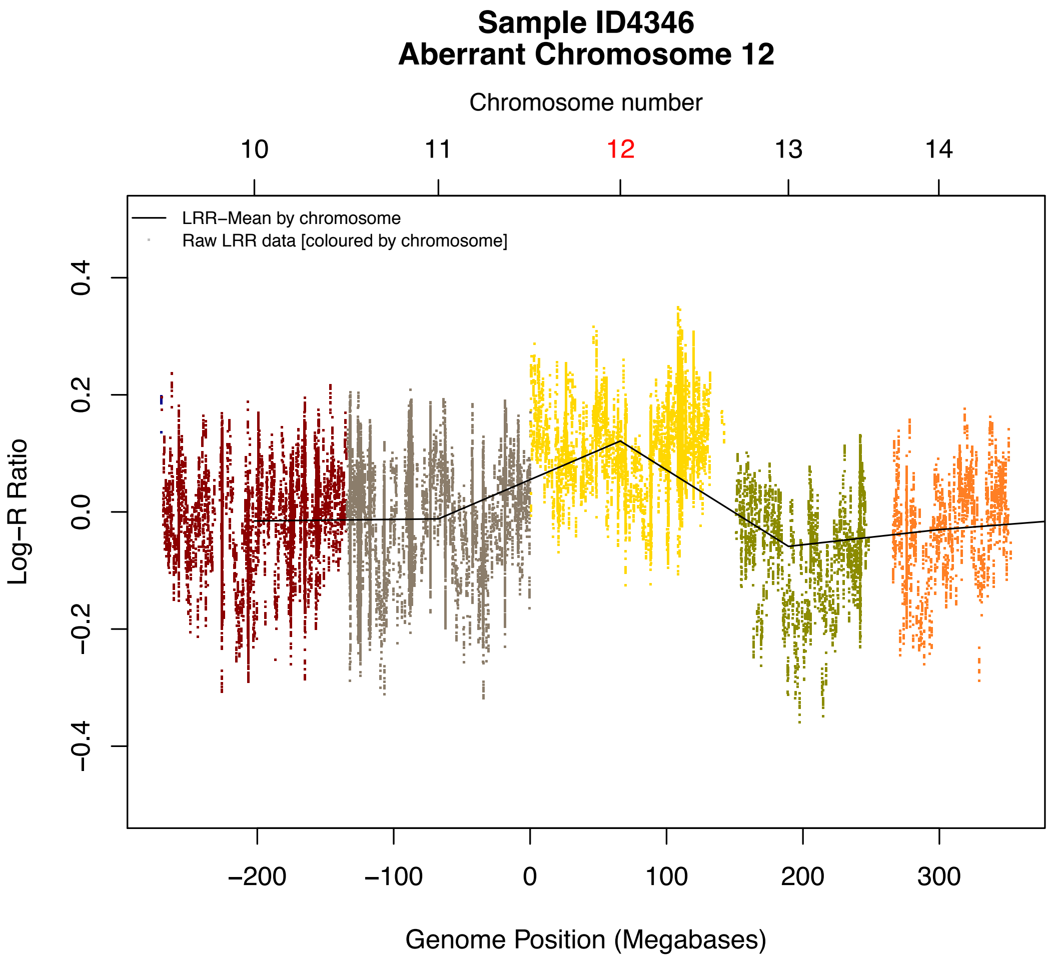

Supplementary Figure 7: *Chromosomal abnormality detection (aneuploidy)*
An example of a clear chromosomal abnormality where the sample appears to have a trisomy 12 mutation. LRR plot of chromosome means 10-14. To preserve sample anonymity the sample ID is a fake id for display purposes only and is not connectable to any record.

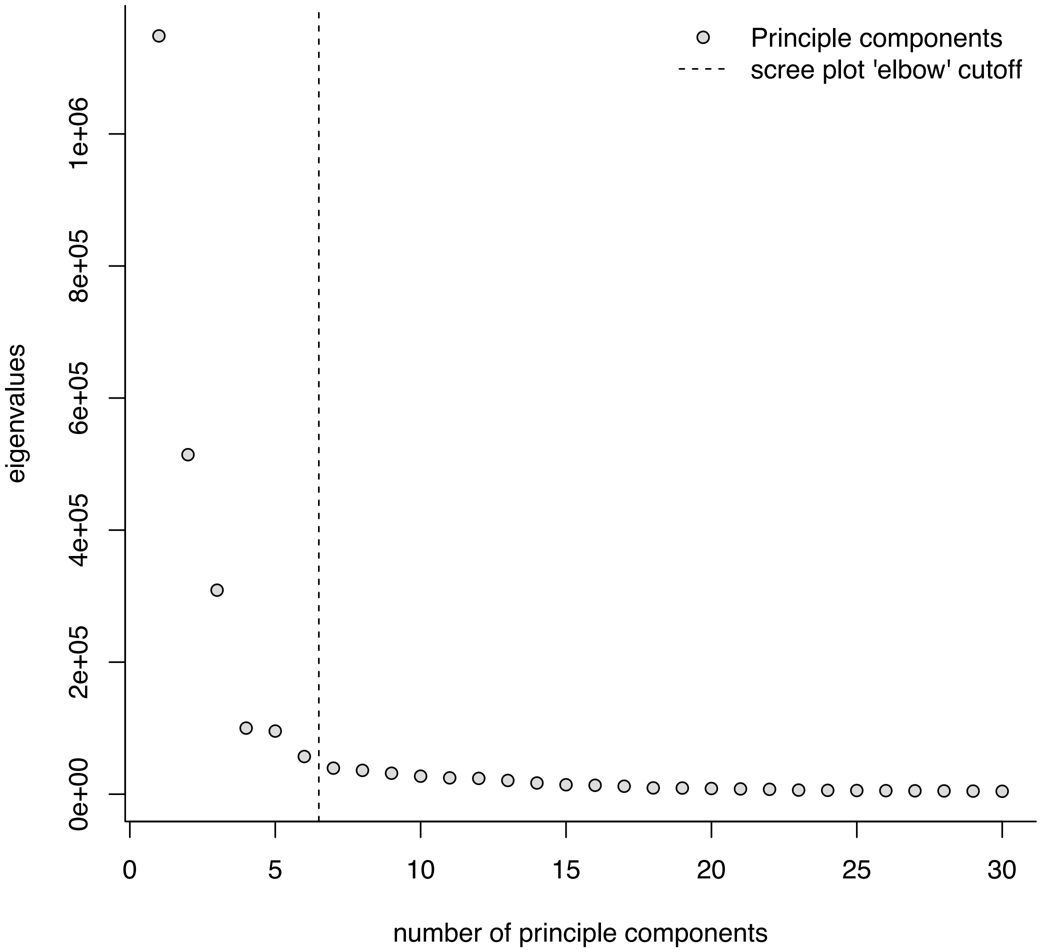

Supplementary Figure 8: Scree plot for PCA: Scree plot of eigenvalues for the PCA on samples passing QC. The vertical dotted line in the scree plot shows a manually identified `elbow' of the plot, a heuristic used to choose a pragmatic set of PCs to use for correction.

Supplementary Table 1. Log R Ratio (LRR) quality control (QC) metrics and sample exclusion summary in T1D cases and controls and families, before and after exclusions and PC correction. Outlier thresholds for the DLSR metric (point-to-point consistency or noise level), absolute GC wave content, and LRR mean, were computed separately for each of the three genotyped datasets (Sanger-genotyped controls, UVA-genotyped controls, and UVA-genotypes cases) and family dataset.

CASE-CONTROL SANGER CONTROLS DATASET, N=4,537

	Mean	DLRS	GC-Wave
Mean	0.0237	0.171	-0.043
SD	0.0199	0.0464	0.0252
Min	-0.0678	0.0995	-0.1038
Q1	0.0114	0.1482	-0.0583
Median	0.0252	0.1638	-0.0448
Q3	0.0371	0.1815	-0.0323
Max	0.1038	0.9285	0.0825
LB	-0.0271	0.0982	-0.0972
UB	0.0756	0.2314	0.0065
-2SD	-0.0162	0.0782	-0.0934
+2SD	0.0635	0.2638	0.0074
-3.5SD	-0.046	0.0087	-0.1312
+3.5SD	0.0934	0.3333	0.0452
Low1%	-0.0292	0.1122	-0.086
Hi1%	0.0692	0.3595	0.0481

CASE-CONTROL UVA T1D DATASET, N=6,808

	Mean	DLRS	GC-Wave
Mean	0.0303	0.1856	-0.0597
SD	0.0196	0.0225	0.0145
Min	-0.1025	0.1159	-0.1527
Q1	0.0198	0.1706	-0.0691
Median	0.0319	0.1855	-0.0598
Q3	0.0438	0.1996	-0.0506
Max	0.0875	0.4055	0.1128
LB	-0.0162	0.1271	-0.0969
UB	0.0798	0.2431	-0.0227
-2SD	-0.0089	0.1406	-0.0886
+2SD	0.0694	0.2306	-0.0307
-3.5SD	-0.0383	0.1068	-0.1103
+3.5SD	0.0988	0.2644	-0.0091
Low1%	-0.0295	0.1376	-0.0925
Hi1%	0.0649	0.2398	-0.0286

Supplementary Table 1 continued.

CASE-CONTROL UVA CONTROLS DATASET, N=5,461 (1958BC)

	Mean	DLRS	GC-Wave
Mean	0.0166	0.1629	-0.0374
SD	0.0191	0.0358	0.0144
Min	-0.1104	0.1129	-0.0876
Q1	0.0064	0.1435	-0.0452
Median	0.0174	0.1562	-0.0368
Q3	0.0272	0.1723	-0.0296
Max	0.0823	0.5598	0.1276
LB	-0.0248	0.1003	-0.0685
UB	0.0584	0.2155	-0.0063
-2SD	-0.0215	0.0914	-0.0663
+2SD	0.0547	0.2344	-0.0086
-3.5SD	-0.0501	0.0377	-0.0879
+3.5SD	0.0833	0.2881	0.0131
Low1%	-0.0382	0.1233	-0.0716
Hi1%	0.0623	0.3112	0.0231

COMBINED CASE-CONTROL DATASET, N=15,900 ; QC-EXCLUSIONS APPLIED AND PC-CORRECTED

	Mean	DLRS	GC-Wave
Mean	0	0.0784	-0.0018
SD	0.0017	0.0186	0.0118
Min	-0.0139	0.0575	-0.0395
Q1	-0.001	0.069	-0.0114
Median	1.00E-04	0.0742	-0.0098
Q3	0.0011	0.0814	0.011
Max	0.0081	0.2873	0.0448
LB	-0.0042	0.0505	-0.0451
UB	0.0043	0.1	0.0446
-2SD	-0.0035	0.0412	-0.0253
+2SD	0.0035	0.1156	0.0218
-3SD	-0.0052	0.0226	-0.0371
+3SD	0.0052	0.1342	0.0335
Low1%	-0.0047	0.0618	-0.0178
Hi1%	0.0039	0.1602	0.0178

Supplementary Table 1 continued.

FAMILIES T1D and UNAFFECTED DATASET, N= 13,070 (T1DGC-ASP)

	Mean	DLRS	GC-Wave
Mean	0.0158	0.174	-0.04
SD	0.0163	0.0414	0.0151
Min	-0.1197	0.1092	-0.1527
Q1	0.0079	0.1543	-0.0483
Median	0.0178	0.1667	-0.0397
Q3	0.0265	0.1812	-0.0322
Max	0.0653	0.7454	0.106
LB	-0.02	0.1139	-0.0725
UB	0.0545	0.2216	-0.0079
-2SD	-0.0168	0.0912	-0.0701
+2SD	0.0483	0.2568	-0.0098
-3.5SD	-0.0412	0.029	-0.0928
+3.5SD	0.0728	0.319	0.0128
Low1%	-0.0396	0.1304	-0.0722
Hi1%	0.0439	0.3675	0.0274

FAMILIES T1D and UNAFFECTED DATASET, N=12,147 ; QC-EXCLUSIONS APPLIED AND PC-CORRECTED

	Mean	DLRS	GC-Wave
Mean	0	0.0802	-0.0012
SD	0.0017	0.0268	0.0116
Min	-0.0107	0.0563	-0.0461
Q1	-0.001	0.0679	-0.0111
Median	0	0.0734	-0.0095
Q3	0.0011	0.0821	0.0109
Max	0.0091	0.3416	0.052
LB	-0.0042	0.0465	-0.0442
UB	0.0043	0.1035	0.044
-2SD	-0.0033	0.0267	-0.0244
+2SD	0.0033	0.1338	0.022
-3SD	-0.005	-1.00E-04	-0.036
+3SD	0.005	0.1606	0.0336
Low1%	-0.0042	0.0603	-0.017
Hi1%	0.004	0.2117	0.017

Note. * Sample metric measures across autosomal chromosomes, before LRR PC-correction; Mean DLRS value across all chromosomes is given as DLRS. T1D = type 1 diabetes; LB = lower bound threshold = Q1-1.5*IQR; Q1 = first quadrant; Q3 = third quadrant; UB = upper bound threshold = Q3+1.5*IQR; IQR = interquartile range; S.D. = standard deviation; Min =minimum value across all samples; Max = maximum value across all samples.

Supplementary Table 2a. Summary of T1D cases and controls that failed one or more QC metric and of the total number of samples submitted for burden of DEL tests.

QC Metric	UVA T1D cases 6,808	UVA controls 5,461	Sanger controls 4,537	Total Samples/SNPs 16,806	Percentage of Total Samples/SNPs
**SNP QC**					
Monomorphic SNPs (NOT EXCLUDED)				22,500	11.45%
SNPs failing HWE p<10^-4				31,106	15.83%
SNPs failing call rate < 0.97				11,945	6.08%
SNPs failing call rate and HWE				9,698	4.93%
SNPs fail t-test between phenotypes post QC				1,937	1.22%
SNPs pass QC				**159,049**	**80.93%**
Samples with genotype CR < 0.94	59	89	97	245	1.45%
Samples with heterozygosity outside 0.1,0.4				3	0.02%
**LRR QC**					
DLRS	32	69	58	159	3.7%
GC Wave	30	100	58	188	0.9%
Chr. Aberrations	182	79	96	357	1.5%
LRR Mean	42	31	9	82	3.0%
Plate QC (>40% samples in a plate fail on above)	0	175	0	175	
Total samples excluded based on LRR QC*	284	436	324	1,044	6.2%
% LRR QC Excluded Samples	4.2%	8.0%	7.1%		
**Samples that passed LRR and Genotype QC**	**6,524**	**5,025**	**4,213**	**15,762**	**93.8%**
**PC-correction**					
SNPs excluded from PCA as part of MHC, centromeres, telomeres, Immunoglobin regions				135	
**Number of SNPs used to run PCA**				25,834	16.24%
**Number of samples used for PCA**				15,762	
**Variance explained by 24 PCs**					95.9%

Note * Sample exclusions based on LRR QC tests are not independent measures, i.e. the total sample failure is not equivalent to the sum of all sample QC failures, as several samples have failed more than one LRR QC; ** Additional exclusions were made based on sex mismatch, sample duplicates, and non-Caucasian ancestry; ^ Plate QC test excluded 3 plates for which the sample failure percentage was above 40% per plate.

Supplementary Table 2b. Summary of T1D cases and unaffected in families that failed one or more QC metrics and of the total number of samples submitted for burden of DEL tests.

**QC Metric**	**Samples/SNPs 13,070**	**Percentage of Samples/SNPs**
**SNP QC**		
Samples with genotype CR < 0.95	324	1.45%
Monomorphic SNPs (NOT EXCLUDED)	22,500	11.45%
SNPs failing call rate < 0.97	17,955	9.14%
SNPs failing HWE p<10^-4 but not call rate	15,918	8.1%
SNPs pass QC	**162,651**	**82.76%**
**LRR QC**		
DLRS	215	1.6%
GC Wave	218	1.7%
Chr. Aberrations	390	3.0%
LRR Mean	120	0.9%
Plate QC (>40% samples in a plate fail on above)	73	0.6%
Total samples excluded based on LRR QC*	1,204	9.2%
**Samples that passed LRR and Genotype QC**	**11,866**	**91.8%**
**PC-correction**		
SNPs excluded from PCA as part of MHC, centromeres, telomeres, Immunoglobin regions	135	
**Number of SNPs used to run PCA**	24,442	15.02%
**Number of samples used for PCA**	11,866	
**Variance explained by 24 PCs**		93.3%
By Phenotype	CASES	CONTROLS
**Final Set of Samples included in TDT analysis Case : Control Samples**	5,892	5,846
% Samples excluded	9.28%	9.92%

Note * Sample exclusions based on LRR QC tests are not independent measures, i.e. the total sample failure is not equivalent to the sum of all sample QC failures, as several samples have failed more than one LRR QC; ** Additional exclusions were made based on sex mismatch, sample duplicates, and non-Caucasian ancestry; ^ Plate QC test excluded 7 plates for which the sample failure percentage was above 40% per. Note that because the family dataset was larger than can be handled by a SnpMatrix object (R-snpStats package), QC was done in plumbCNV via Plink which did not provide separate counts for HWE/Call-rate and did not provide sample-heterozygosity calculations, and so this is why the information in the SNPQC rows differs slightly versus the case-control version of the same table.

Supplementary Table 2c. Summary of CNV QC exclusions for the case-control and family datasets.

CASE-CONTROL

CNV QC	All CNVs	rDUPs	rDELs	Plate	Total CNV
CNV-based Sample Exclusions	*94*	*33*	*4*	0 CNP +24 CNV	*155*
Consequent number of CNVs excluded	*1,889 CNP*	*245*	*30*	0 CNP +69 CNV	*344*
CNV Length Sample Exclusion (> 5 Mb)		1	4		5
CNVs passing quality score cutoff (rDUP>.75, rDEL>.95)		*4,676/5,295*	*2,432/2,936*		

FAMILIES

CNV QC	All CNVs	rDUPs	rDELs	Plate	Total CNV
CNV-based Sample Exclusions	*35*	*43*	*1*	31 CNP +57 CNV	*101*
Consequent number of CNVs excluded	*772 CNP*	*322*	*8*	138 CNP +182 CNV	*512*
CNV Length Sample Exclusion (> 5 Mb)		4	2		6
CNVs passing quality score cutoff (rDUP>.75, rDEL>.95)		*3,055/3,373*	*2,861/3,431*		

Note * Samples were excluded that had too many overall CNVs (i.e CNVs + CNPs) because this is an indicator of poor data quality. The 'total CNV' column shows only sample and CNV counts of excluded rare CNVs. The thresholds for rDUP exclusion are more lax than for rDEL exclusion, as validation of the same samples in a different dataset (Supplementary Methods section 5) showed these to be optimal thresholds. Note that the length sample exclusion is only used for overall burden and gene/exon overlap tests, not for analysis of length.

Supplementary Table 3. LRR-based sample failure relationship between different QC metrics. Each cell value is the number of samples that failed on both the column metric and the row metric, so for instance the diagonal shows the totals for each type ignoring overlap. The final row shows the counts for samples that fail *only* on the column metric and no other. Overall, there is fair independence between tests, suggesting that each test captures separate aspects of LRR distributions.

CASE-CONTROL DATASET: LRR Sample QC-exclusions

	Mean	DLRS	ChrAb	GC-Wave
Mean	82	26	39	21
DLRS	26	159	38	6
ChrAb	39	38	357	114
GCWave	21	6	114	188
No Others	29	109	204	70

FAMILIES DATASET: LRR Sample QC-exclusions

	Mean	DLRS	ChrAb	GC-Wave
Mean	120	34	27	29
DLRS	34	215	32	17
ChrAb	27	32	390	122
GCWave	29	17	122	218
No Others	59	155	244	83

Note: DLRS = Derivative Log Ratio Spread, ChrAb. = chromosomal aberrations, LRR = Log R Ratio

Supplementary Table 4. rare DELs in the case-control study exceeding 400 kb.

locus	start (bp)	end (bp)	length (bp)	n-sites	phenotype	genes
1p22.3	87,235,343	88,004,704	769,362	16	T1D	*HS2ST1,LMO4*
1q23.1	155,603,560	156,527,639	924,080	34	T1D	*FCRL5,FCRL4,FCRL3,FCRL2,FCRL1,CD5L,KIRREL,CD1D, RPS10P8,CD1A,CD1C*
1q23.1-1q23.2	157,245,220	158,039,375	794,156	23	T1D	*IFI16,AIM2,CADM3,DARC,OR10J5,FCER1A,OR10J3,OR10J 1,OR10J9P,APCS,CRP,DUSP23,FCRL6*
1q41	215,128,763	216,794,613	1,665,851	33	Control	*ESRRG,GPATCH2,SPATA17,UBBP2,RRP15,TGFB2*
1q41	215,128,763	216,794,613	1,665,851	33	Control	*ESRRG,GPATCH2,SPATA17,UBBP2,RRP15,TGFB2*
1q43	237,142,337	239,237,790	2,095,454	50	Control	*CHRM3,RPS7P5,FMN2,ADH5P3,GREM2,RGS7*
1q43	240,381,977	241,112,054	730,078	421	Control	*PLD5*
2p23.3	25,003,620	25,414,511	410,892	167	Control	*DNAJC27,EFR3B,POMC,DNMT3A,MIRN1301*
2p22.1-2p21	40,145,042	43,305,838	3,160,797	250	T1D	*SLC8A1,LDHAL3,RPS12P4,EML4,COX7A2L,KCNG3,MTA3, OXER1,HAAO,ZFP36L2*
2p21	44,131,231	44,754,350	623,120	12	Control	*PPM1B,SLC3A1,PREPL,C2orf34*
2q12.2-2q12.3	106,469,122	107,794,449	1,325,328	13	T1D	*CD8BP,ST6GAL2*
2q12.2-2q12.3	106,469,122	107,794,449	1,325,328	13	T1D	*CD8BP,ST6GAL2*
2q12.2-2q12.3	106,469,122	107,473,982	1,004,861	12	Control	*CD8BP,ST6GAL2*
2q13	111,331,550	112,805,738	1,474,189	38	T1D	*ACOXL,BCL2L11,ANAPC1,MERTK,TMEM87B,FBLN7,ZC3H 8,ZC3H6*
2q13	112,180,515	112,805,738	625,224	20	T1D	*ANAPC1,MERTK,TMEM87B,FBLN7,ZC3H8,ZC3H6*
2q14.3	126,498,456	127,240,554	742,099	15	T1D	*GYPC*
2q23.3	153,172,826	153,696,621	523,796	11	Control	*FMNL2,PRPF40A,ARL6IP6*

2q37.3	238,672,730	242,442,790	3,770,061	288	T1D	*SCLY,ESPNL,KLHL30,FAM132B,ILKAP,HES6,PER2,TRAF3IP1,ASB1,HDAC4,NDUFA10,OR6B2,OR6B3,OR9S24P,MYEOV2,OTOS,GPC1,MIRN149,ANKMY1,DUSP28,RNPEPL1,CAPN10,GPR35,AQP12B,AQP12A,KIF1A,AGXT,C2orf54,SNED1,MTERFD2,PASK,PPP1R7,ANO7,HDLBP,SEPT2,FARP2,STK25,BOK,THAP4,ATG4B,DTYMK,ING5,D2HGDH,GAL3ST2,NEU4,PDCD1*
3p26.3	37,614	1,318,376	1,280,763	21	Control	*CHL1,CNTN6*
3p26.3	376,533	1,383,344	1,006,812	16	T1D	*CHL1,CNTN6*
3p26.3	1,312,371	1,836,001	523,631	20	Control	*CNTN6,RPL23AP38*
3p26.3	1,312,371	2,323,159	1,010,789	25	Control	*CNTN6,RPL23AP38,CNTN4*
3p26.3	1,836,001	2,328,515	492,515	8	Control	*CNTN4*
3p26.3	2,120,996	2,930,529	809,534	19	T1D	*CNTN4*
3p26.3	2,120,996	2,930,529	809,534	19	Control	*CNTN4*
3p26.3-3p26.2	2,989,060	4,969,471	1,980,412	42	T1D	*CNTN4,IL5RA,TRNT1,CRBN,LRRN1,SUMF1,SETMAR,ITPR1*
3p21.1	53,721,246	54,290,074	568,829	44	Control	*CACNA1D,CHDH,IL17RB,ACTR8,CACNA2D3*
3p12.3	75,816,778	77,017,391	1,200,614	13	Control	*ROBO2*
3p12.3	76,074,141	78,753,344	2,679,204	24	T1D	*ROBO2,ROBO1*
3p12.2-3p11.2	83,006,051	87,411,438	4,405,388	38	T1D	*CADM2,VGLL3,CHMP2B,POU1F1*
3q25.1	151,215,986	152,757,036	1,541,051	19	T1D	*TSC22D2,SERP1,EIF2A,C3orf44,SIAH2,C3orf76,CLRN1,MED12L,GPR171,P2RY14,GPR87,P2RY13,P2RY12,IGSF10*
3q25.1-3q25.2	153,073,120	154,155,428	1,082,309	52	Control	*SUCNR1,MBNL1,TMEM14E,P2RY1*
3q26.1	165,055,312	166,683,541	1,628,230	23	Control	*MIRN1263,MIRN720,SI,SLITRK3*
3q28-3q29	193,336,343	196,623,883	3,287,541	34	T1D	*FGF12,C3orf59,HRASLS,ATP13A5,ATP13A4,OPA1,HES1,CPN2,LRRC15,GP5,ATP13A3,TMEM44,LSG1,FAM43A,C3orf21,ACAP2*
4p15.1	31,387,582	32,598,277	1,210,696	13	T1D	*intergenic*
4p15.1	31,387,582	32,598,277	1,210,696	13	T1D	*intergenic*
4p15.1	31,387,582	32,598,277	1,210,696	13	T1D	*intergenic*
4q12-4q13.2	58,527,617	66,527,108	7,999,492	83	T1D	*LPHN3,EPHA5*
4q21.22-4q21.23	84,277,251	85,334,034	1,056,784	13	Control	*COQ2,HPSE,HELQ,MRPS18C,FAM175A,AGPAT9*

4q22.1	90,629,574	91,244,873	615,300	33	Control	*SNCA,MMRN1*
4q22.1	90,692,563	91,244,873	552,311	32	Control	*SNCA,MMRN1*
4q22.1-4q22.3	93,619,076	95,834,792	2,215,717	17	Control	*GRID2,ATOH1,SMARCAD1,PDLIM5*
4q28.3	138,327,813	139,014,722	686,910	17	Control	*PCDH18*
4q32.3	166,482,497	166,885,930	403,434	13	Control	*SC4MOL,CPE,MIRN578*
4q35.2	188,313,710	189,096,902	783,193	20	Control	*intergenic*
4q35.2	188,313,710	189,096,902	783,193	20	Control	*intergenic*
4q35.2	188,705,876	189,985,301	1,279,426	27	Control	*ZFP42,TRIML2,TRIML1*
4q35.2	188,961,800	189,759,104	797,305	20	T1D	*ZFP42,TRIML2,TRIML1*
4q35.2	189,453,783	189,985,301	531,519	11	Control	*intergenic*
4q35.2	189,453,783	189,985,301	531,519	11	T1D	*intergenic*
4q35.2	189,613,473	190,980,780	1,367,308	19	Control	*HSP90AA4P*
5q15	95,743,375	96,794,354	1,050,980	1,213	Control	*PCSK1,CAST,ERAP1,ERAP2,LNPEP,LIX1,RIOK2*
6q12	67,441,289	67,889,552	448,264	10	Control	*intergenic*
6q15-6q16.1	91,512,205	94,465,435	2,953,231	40	Control	*EPHA7*
6q15-6q16.1	91,512,205	94,465,435	2,953,231	40	T1D	*EPHA7*
6q16.1-6q22.31	97,549,988	121,050,378	23,500,391	1,659	T1D	*KLHL32,C6orf167,POU3F2,FBXL4,C6orf168,COQ3,SFRS18,USP45,CCNC,PRDM13,MCHR2,SIM1,ASCC3,GRIK2,HACE1,LIN28B,BVES,C6orf112,POPDC3,PREP,RPL35P3,PRDM1,ATG5,AIM1,RTN4IP1,QRSL1,MIRN587,C6orf203,BEND3,PDSS2,SOBP,SCML4,SEC63,RPL3P7,OSTM1,NR2E1,SNX3,LACE1,FOXO3,ZNF259P,ARMC2,SESN1,C6orf182,C6orf183,C6orf184,C6orf185,CD164,PPIL6,SMPD2,MICAL1,ZBTB24,C6orf224,AKD1,AKD2,FIG4,GPR6,WASF1,CDC40,C6orf186,DDO,SLC22A16,CDC2L6,AMD1,GTF3C6,BXDC1P,BXDC1,SLC16A10,KIAA1919,REV3L,TRAF3IP2,FYN,WISP3,TUBE1,C6orf225,LAMA4,RFPL4B,RPSAP45,RPL30P8,MARCKS,HDAC2,HS3ST5,FRK,NT5DC1,COL10A1,TSPYL4,TSPYL1,DSE,FAM26F,BET3L,FAM26E,FAM26D,RWDD1L1,RWDD1,RSPH4A,ZUFSP,KPNA5,FAM162B,GPRC6A,RFX6,VGLL2,ROS1,DCBLD1,GOPC,NUS1,SLC35F1,C6orf204,BRD7P3,PLN,MCM9,ASF1A,FAM184A,MIRN548B,MAN1A1*
6q23.3	135,761,193	136,175,352	414,160	11	T1D	*AHI1*
7p21.3	9,834,067	10,791,300	957,234	30	Control	*intergenic*
7q21.13	88,386,077	88,872,879	486,803	13	T1D	*ZNF804B*
7q21.13	89,922,392	90,556,222	633,831	20	T1D	*PFTK1*

7q35	144,155,157	145,212,015	1,056,859	10	Control	*TPK1*
7q35	144,155,157	145,212,015	1,056,859	10	Control	*TPK1*
8p23.3	207,587	859,170	651,584	12	Control	*FAM87A,FBXO25,C8orf42,ERICH1*
8p23.2	5,148,034	5,903,912	755,879	25	T1D	*intergenic*
8p23.2	5,148,034	5,633,607	485,574	16	T1D	*intergenic*
8p23.2	5,428,726	6,014,266	585,541	25	T1D	*intergenic*
8p22	13,118,507	14,128,982	1,010,476	25	Control	*DLC1,C8orf48,SGCZ*
8p22	14,243,385	16,311,380	2,067,996	31	T1D	*SGCZ,MIRN383,TUSC3,MSR1*
8q21.13	83,429,776	84,883,781	1,454,006	25	Control	*intergenic*
8q24.21-8q24.22	131,199,776	133,611,256	2,411,481	50	T1D	*ASAP1,ADCY8,EFR3A,OC90,KCNQ3*
8q24.22	132,187,347	132,633,840	446,494	12	Control	*intergenic*
9p23	11,329,109	12,346,017	1,016,909	11	Control	*intergenic*
9p22.2	17,213,492	17,897,828	684,337	9	Control	*CNTLN,SH3GL2*
9p21.2-9p21.1	27,755,416	28,180,379	424,964	17	T1D	*LINGO2*
10q11.22-10q11.23	47,985,120	51,290,323	3,305,204	650	Control	*ZNF488,RBP3,GDF2,GDF10,PTPN20B,FRMPD2L1,CTGLF9P,BMS1P5,GLUDP2,CTSLL3,FAM25G,FAM25B,FAM25C,BMS1P7,FRMPD2,MAPK8,ARHGAP22,WDFY4,RPL13AP19,LRRC18,C10orf72,FAM170B,C10orf128,C10orf71,DRGX,ERCC6,PGBD3,CHAT,SLC18A3,C10orf53,OGDHL,PARG,FAM25D,FAM25E,AGAP8,TIMM23B,AGAP7,RPL23AP61,MSMB,NCOA4,TIMM23*
10q11.22-10q11.23	47,985,120	51,290,323	3,305,204	650	Control	*ZNF488,RBP3,GDF2,GDF10,PTPN20B,FRMPD2L1,CTGLF9P,BMS1P5,GLUDP2,CTSLL3,FAM25G,FAM25B,FAM25C,BMS1P7,FRMPD2,MAPK8,ARHGAP22,WDFY4,RPL13AP19,LRRC18,C10orf72,FAM170B,C10orf128,C10orf71,DRGX,ERCC6,PGBD3,CHAT,SLC18A3,C10orf53,OGDHL,PARG,FAM25D,FAM25E,AGAP8,TIMM23B,AGAP7,RPL23AP61,MSMB,NCOA4,TIMM23*
10q11.22-10q11.23	49,171,289	50,579,296	1,408,008	641	T1D	*MAPK8,ARHGAP22,WDFY4,RPL13AP19,LRRC18,C10orf72,FAM170B,C10orf128,C10orf71,DRGX,ERCC6,PGBD3,CHAT,SLC18A3,C10orf53*
10q21.1	54,224,030	56,883,032	2,659,003	139	Control	*PCDH15,MIRN548F1*
10q21.1	55,502,506	56,361,311	858,806	15	T1D	*PCDH15,MIRN548F1*

10q22.3-10q23.2	81,641,502	88,935,321	7,293,820	136	T1D	*SFTPD,PLAC9,ANXA11,MAT1A,DYDC1,DYDC2,C10orf57,C10orf58,TSPAN14,SH2D4B,NRG3,GHITM,C10orf99,PCDH21,LRIT2,LRIT1,RGR,KIAA1128,GRID1,MIRN346,WAPAL,RPL7AP8,OPN4,LDB3,BMPR1A,MMRN2,SNCG,C10orf116,AGAP11,FAM25A,FAM25G,FAM25B,FAM25C,GLUD1,FAM35A*
10q26.3	134,144,849	135,040,419	895,571	24	T1D	*INPP5A,NKX6-2,C10orf92,C10orf93,GPR123,KNDC1,UTF1,VENTX,MIRN202,ADAM8,TUBGCP2,ZNF511,CALY,PRAP1,C10orf125,ECHS1*
11p14.3	24,556,454	25,820,126	1,263,673	23	T1D	*LUZP2*
11p14.3-11p14.2	25,524,245	26,627,937	1,103,693	30	T1D	*ANO3,MUC15*
11q14.1	82,954,144	83,656,956	702,813	16	T1D	*DLG2*
11q24.3-11q25	128,260,474	134,268,351	6,007,878	100	T1D	*KCNJ5,C11orf45,TP53AIP1,BARX2,TMEM45B,NFRKB,PRDM10,APLP2,ST14,ZBTB44,ADAMTS8,ADAMTS15,C11orf44,SNX19,NTM,OPCML,SPATA19,IGSF9B,JAM3,NCAPD3,VPS26B,THYN1,ACAD8,GLB1L3,GLB1L2,B3GAT1*
12p13.33	534,755	2,535,198	2,000,444	408	Control	*B4GALNT3,NINJ2,WNK1,RAD52,ERC1,FBXL14,WNT5B,ADIPOR2,CACNA2D4,LRTM2,DCP1B,CACNA1C*
12q24.33	129,709,315	132,228,483	2,519,169	47	T1D	*STX2,RAN,GPR133,SFRS8,MMP17,ULK1,PUS1,EP400,SNORA49,EP400NL,DDX51,NOC4L,GALNT9,MUC8,FBRSL1,P2RX2,POLE,PXMP2,PGAM5,ANKLE2,GOLGA3,CHFR,ZNF605,ZNF26,ZNF84,ZNF140,ZNF10*
13q12.12	22,448,723	23,696,995	1,248,273	36	Control	*HMGA1L6,SGCG,SACS,TNFRSF19,MIPEP,SPATA13*
13q12.12	22,448,723	23,696,995	1,248,273	36	T1D	*HMGA1L6,SGCG,SACS,TNFRSF19,MIPEP,SPATA13*
13q12.12	22,448,723	23,696,995	1,248,273	36	T1D	*HMGA1L6,SGCG,SACS,TNFRSF19,MIPEP,SPATA13*
13q12.12	22,448,723	23,696,995	1,248,273	36	T1D	*HMGA1L6,SGCG,SACS,TNFRSF19,MIPEP,SPATA13*
13q12.12	22,448,723	23,696,995	1,248,273	36	Control	*HMGA1L6,SGCG,SACS,TNFRSF19,MIPEP,SPATA13*
13q12.12	22,695,713	23,696,995	1,001,283	26	T1D	*SGCG,SACS,TNFRSF19,MIPEP,SPATA13*
13q12.12	23,101,027	23,525,618	424,592	10	T1D	*TNFRSF19,MIPEP*

13q14.11	41,094,470	41,551,437	456,968	10	Control	*KIAA0564,DGKH*
13q33.1-13q33.2	102,434,239	103,742,464	1,308,226	24	Control	*SLC10A2*
14q21.2-14q21.3	42,452,112	43,248,804	796,693	20	Control	*intergenic*
14q21.3	43,248,804	44,316,771	1,067,968	14	Control	*FSCB*
14q31.1-14q31.2	79,635,167	83,717,917	4,082,751	1,276	T1D	*DIO2,C14orf145,TSHR,GTF2A1,SNORA79,STON2,SEL1L*
14q32.32-14q32.33	102,748,906	104,688,936	1,940,031	87	Control	*EIF5,SNORA28,MARK3,CKB,TRMT61A,BAG5,C14orf153,KLC1,XRCC3,ZFYVE21,PPP1R13B,C14orf2,TDRD9,ASPG,MIRN203,KIF26A,C14orf180,TMEM179,INF2,ADSSL1,SIVA1,AKT1,RPS2P4,KIAA0284,PLD4,AHNAK2,C14orf79,CDCA4,GPR132,JAG2*
15q13.1-15q13.2	27,081,472	28,148,879	1,067,408	22	Control	*APBA2,NDNL2,TJP1*
15q13.1-15q13.2	27,186,889	28,148,879	961,991	21	Control	*APBA2,NDNL2,TJP1*
15q13.2-15q13.3	28,737,821	30,198,807	1,460,987	42	T1D	*ARHGAP11B,MTMR15,MTMR10,TRPM1,MIRN211,KLF13,OTUD7A,CHRNA7*
15q13.2-15q13.3	28,737,821	30,198,807	1,460,987	42	T1D	*ARHGAP11B,MTMR15,MTMR10,TRPM1,MIRN211,KLF13,OTUD7A,CHRNA7*
15q13.2-15q13.3	28,737,821	30,198,807	1,460,987	42	Control	*ARHGAP11B,MTMR15,MTMR10,TRPM1,MIRN211,KLF13,OTUD7A,CHRNA7*
15q13.2-15q13.3	28,737,821	30,198,807	1,460,987	42	T1D	*ARHGAP11B,MTMR15,MTMR10,TRPM1,MIRN211,KLF13,OTUD7A,CHRNA7*
15q25.2	81,023,980	82,491,787	1,467,808	21	Control	*CPEB1,AP3B2,SCARNA15,FSD2,WHAMM,HOMER2,FAM103A1,C15orf40,BTBD1,TM6SF1,BNC1,SH3GL3,ADAMTSL3*
15q26.2	92,849,065	93,891,156	1,042,092	18	T1D	*intergenic*
16p13.3-16p13.2	6,192,218	6,802,012	609,795	23	T1D	*intergenic*
16p13.2	6,351,729	6,802,012	450,284	19	T1D	*intergenic*
16p13.2	6,488,390	7,002,554	514,165	18	Control	*intergenic*
16p13.11-16p12.3	15,726,343	17,930,955	2,204,613	26	T1D	*NDE1,MYH11,C16orf63,ABCC1,ABCC6,NOMO3,XYLT1*
16p13.11-16p12.3	15,726,343	17,930,955	2,204,613	26	T1D	*NDE1,MYH11,C16orf63,ABCC1,ABCC6,NOMO3,XYLT1*
16p11.2	28,393,346	28,933,479	540,134	312	T1D	*CLN3,IL27,CCDC101,SULT1A2,SULT1A1,EIF3C,ATXN2L,TUFM,SH2B1,ATP2A1,RABEP2,CD19,NFATC2IP,SPNS1,LAT*

16p11.2	28,394,557	28,933,479	538,923	311	T1D	*CLN3,IL27,CCDC101,SULT1A2,SULT1A1,EIF3C,ATXN2L,TUFM,SH2B1,ATP2A1,RABEP2,CD19,NFATC2IP,SPNS1,LAT*
16p11.2	29,600,193	30,095,177	494,985	17	Control	*QPRT,C16orf54,KIF22,MAZ,PRRT2,C16orf53,MVP,CDIPT,SEZ6L2,ASPHD1,KCTD13,TMEM219,TAOK2,HIRIP3,INO80E,DOC2A,FAM57B,ALDOA,PPP4C,TBX6,ZNF785,ZNF688,YPEL3,GDPD3,MAPK3*
16p11.2	29,600,193	30,095,177	494,985	17	Control	*QPRT,C16orf54,KIF22,MAZ,PRRT2,C16orf53,MVP,CDIPT,SEZ6L2,ASPHD1,KCTD13,TMEM219,TAOK2,HIRIP3,INO80E,DOC2A,FAM57B,ALDOA,PPP4C,TBX6,ZNF785,ZNF688,YPEL3,GDPD3,MAPK3*
16p11.2	29,600,193	30,095,177	494,985	17	T1D	*QPRT,C16orf54,KIF22,MAZ,PRRT2,C16orf53,MVP,CDIPT,SEZ6L2,ASPHD1,KCTD13,TMEM219,TAOK2,HIRIP3,INO80E,DOC2A,FAM57B,ALDOA,PPP4C,TBX6,ZNF785,ZNF688,YPEL3,GDPD3,MAPK3*
16p11.2	29,600,193	30,095,177	494,985	17	Control	*QPRT,C16orf54,KIF22,MAZ,PRRT2,C16orf53,MVP,CDIPT,SEZ6L2,ASPHD1,KCTD13,TMEM219,TAOK2,HIRIP3,INO80E,DOC2A,FAM57B,ALDOA,PPP4C,TBX6,ZNF785,ZNF688,YPEL3,GDPD3,MAPK3*
16p11.2	29,600,193	30,095,177	494,985	17	Control	*QPRT,C16orf54,KIF22,MAZ,PRRT2,C16orf53,MVP,CDIPT,SEZ6L2,ASPHD1,KCTD13,TMEM219,TAOK2,HIRIP3,INO80E,DOC2A,FAM57B,ALDOA,PPP4C,TBX6,ZNF785,ZNF688,YPEL3,GDPD3,MAPK3*
16p11.2	29,600,193	30,095,177	494,985	17	Control	*QPRT,C16orf54,KIF22,MAZ,PRRT2,C16orf53,MVP,CDIPT,SEZ6L2,ASPHD1,KCTD13,TMEM219,TAOK2,HIRIP3,INO80E,DOC2A,FAM57B,ALDOA,PPP4C,TBX6,ZNF785,ZNF688,YPEL3,GDPD3,MAPK3*
16p11.2	29,600,193	30,095,177	494,985	17	T1D	*QPRT,C16orf54,KIF22,MAZ,PRRT2,C16orf53,MVP,CDIPT,SEZ6L2,ASPHD1,KCTD13,TMEM219,TAOK2,HIRIP3,INO80E,DOC2A,FAM57B,ALDOA,PPP4C,TBX6,ZNF785,ZNF688,YPEL3,GDPD3,MAPK3*
16q12.1-16q12.2	50,853,681	51,474,818	621,138	41	Control	*TOX3*
16q23.3	80,830,873	82,216,150	1,385,278	33	Control	*CDH13*
16q23.3	80,830,873	82,216,150	1,385,278	33	Control	*CDH13*
17p13.3-17p13.2	3,011,784	3,677,728	665,945	12	Control	*OR1A2,OR1A1,OR3A2,OR3A1,OR3A4,OR1E1,OR3A3,OR1E2,SPATA22,ASPA,TRPV3,TRPV1,SHPK,CTNS,TAX1BP3,TMEM93,P2RX5,ITGAE,GSG2,C17orf85*
17p13.2	5,567,013	6,176,952	609,940	22	T1D	*WSCD1*
17p13.2	5,567,013	6,176,952	609,940	22	T1D	*WSCD1*

17p12	14,125,873	15,298,132	1,172,260	19	Control	*HS3ST3B1,PMP22,TEKT3,CDRT4,RPL9P2*
17p12	14,125,873	15,323,513	1,197,641	20	Control	*HS3ST3B1,PMP22,TEKT3,CDRT4,RPL9P2*
17p12	14,125,873	15,323,513	1,197,641	20	T1D	*HS3ST3B1,PMP22,TEKT3,CDRT4,RPL9P2*
17p12	14,125,873	15,298,132	1,172,260	19	T1D	*HS3ST3B1,PMP22,TEKT3,CDRT4,RPL9P2*
17p12	14,125,873	15,281,908	1,156,036	18	Control	*HS3ST3B1,PMP22,TEKT3,CDRT4*
17p12	14,125,873	15,281,908	1,156,036	18	Control	*HS3ST3B1,PMP22,TEKT3,CDRT4*
17p12	14,125,873	15,323,513	1,197,641	20	Control	*HS3ST3B1,PMP22,TEKT3,CDRT4,RPL9P2*
17p12	14,125,873	15,323,513	1,197,641	20	T1D	*HS3ST3B1,PMP22,TEKT3,CDRT4,RPL9P2*
17p12	14,125,873	15,323,513	1,197,641	20	Control	*HS3ST3B1,PMP22,TEKT3,CDRT4,RPL9P2*
17q12	31,906,076	33,175,269	1,269,194	12	T1D	*ZNHIT3,MYO19,PIGW,GGNBP2,DHRS11,MRM1,LHX1,AATF,ACACA,C17orf78,TADA2L,DUSP14,AP1GBP1,DDX52,HNF1B*
17q12	31,906,076	33,175,269	1,269,194	12	T1D	*ZNHIT3,MYO19,PIGW,GGNBP2,DHRS11,MRM1,LHX1,AATF,ACACA,C17orf78,TADA2L,DUSP14,AP1GBP1,DDX52,HNF1B*
17q21.31	41,069,708	41,545,191	475,484	44	T1D	*C17orf69,CRHR1,MAPT,KIAA1267*
17q21.31	41,069,708	41,545,191	475,484	44	T1D	*C17orf69,CRHR1,MAPT,KIAA1267*
18p11.32-18p11.31	174,476	5,760,922	5,586,447	103	T1D	*USP14,THOC1,COLEC12,CETN1,CLUL1,C18orf56,TYMS,ENOSF1,YES1,ADCYAP1,C18orf2,METTL4,NDC80,SMCHD1,EMILIN2,LPIN2,MYOM1,MYL12A,MYL12B,TGIF1,DLGAP1,ZFP161,EPB41L3*
18q12.3	35,851,608	36,923,971	1,072,364	35	T1D	*intergenic*
18q12.3	35,851,608	36,923,971	1,072,364	35	T1D	*intergenic*
18q23	71,651,417	76,115,293	4,463,877	85	T1D	*ZNF516,ZNF236,MBP,GALR1,SALL3,ATP9B,NFATC1,CTDP1,KCNG2,PQLC1,TXNL4A,C18orf22,ADNP2,PARD6G*
19p12	21,862,211	22,877,857	1,015,647	9	T1D	*ZNF208,ZNF257,ZNF676,ZNF729,ZNF849P,ZNF492,ZNF99*

20p12.3-20p12.2	8,537,571	9,490,728	953,158	10	Control	*PLCB1,RNU105B,PLCB4,C20orf103,PAK7*
20p12.1	14,014,276	14,630,334	616,059	13	T1D	*MACROD2,SCYE1P,FLRT3*
20p12.1	14,650,391	15,200,715	550,325	13	T1D	*MACROD2,RPS10P2*
20q11.23	34,922,679	35,700,203	777,525	18	T1D	*C20orf117,C20orf118,SAMHD1,RBL1,RPS3AP3,RPS27AP3,C20orf132,RPN2,GHRH,MANBAL,SRC,BLCAP,NNAT,PPIAP3,GLRXP*
20q13.13	45,913,972	46,456,305	542,334	28	Control	*intergenic*
20q13.13	45,913,972	46,420,710	506,739	27	T1D	*intergenic*
21q21.2	24,032,141	25,544,008	1,511,868	28	T1D	*intergenic*
22q11.21	17,527,441	19,792,353	2,264,913	41	Control	*SLC25A1,CLTCL1,HIRA,MRPL40,C22orf39,UFD1L,CDC45L,CLDN5,SEPT5,GP1BB,TBX1,GNB1L,C22orf29,TXNRD2,COMT,ARVCF,C22orf25,MIRN185,DGCR8,MIRN1306,TRMT2A,RANBP1,ZDHHC8,RTN4R,MIRN1286,DGCR6L,GGTLC3,TMEM191C,PI4KAP1,RIMBP3,SUSD2P1,SUSD2P2,USP41,ZNF74,SCARF2,KLHL22,MED15,FAM108A5,POM121L4P,PI4KA,SERPIND1,SNAP29,CRKL,AIFM3,LZTR1,THAP7,P2RX6,SLC7A4,MIRN649,P2RX6P*
22q11.21	19,124,484	19,792,353	667,870	7	T1D	*KLHL22,MED15,FAM108A5,POM121L4P,PI4KA,SERPIND1,SNAP29,CRKL,AIFM3,LZTR1,THAP7,P2RX6,SLC7A4,MIRN649,P2RX6P*
22q13.31	44,732,768	45,499,803	767,036	37	T1D	*WNT7B,C22orf26,MIRNLET7A3,MIRNLET7B,PPARA,C22orf40,PKDREJ,TTC38,GTSE1,TRMU,CELSR1,GRAMD4,CERK*

Supplementary Table 5. Summary of the runs of the CNV pipeline varying QC parameters.

Listing of performance for each of 54 runs when the parameters for Sample QC, SNP QC, PC correction and CNV-QC were varied. Abbreviations: QS=quality score, OR=case:control odds ratio, Sens=sensitivity in replicating DGV CNVs that had sufficient ImmunoChip coverage, Spec=specificity, for CNVs we detected to have previously been found in the DGV; rDEL=rare deletions, rDUP=rare duplications. Based on intersection with all databases for at least 6 sites per CNV.

					**rDEL**				**rDUP**			
Run	*SAMPLE QC*	*SNP QC*	*PCA*	*CNV-QC*	*QS*	*OR*	*Sens*	*Spec*	*QS*	*OR*	*Sens*	*Spec*
1	stringent	medium	24	none	0.91	1.08	0.4	0.45	0.91	1.01	0.41	0.57
2	stringent	medium	24	full	0.95	0.99	0.35	0.45	0.94	1.04	0.39	0.56
3	stringent	medium	6	none	0.91	1.53	0.45	0.43	0.91	0.93	0.44	0.4
4	stringent	medium	6	full	0.95	1.14	0.37	0.45	0.94	1.11	0.4	0.51
5	stringent	medium	0	none	0.62	1.67	0.42	0.46	0.57	1.17	0.54	0.16
6	stringent	medium	0	full	0.72	1	0.33	0.46	0.57	1.5	0.44	0.14
7	stringent	complete	24	none	0.9	1.11	0.4	0.46	0.91	1	0.41	0.57
8	stringent	complete	24	full	0.95	1.01	0.35	0.46	0.94	0.94	0.39	0.56
9	stringent	complete	6	none	0.91	1.48	0.44	0.44	0.9	0.93	0.44	0.4
10	stringent	complete	6	full	0.95	1.16	0.36	0.46	0.93	1.02	0.4	0.5
11	stringent	complete	0	none	0.59	1.66	0.41	0.45	0.57	1.24	0.54	0.17
12	stringent	complete	0	full	0.67	1	0.33	0.45	0.58	1.47	0.45	0.15
13	stringent	none	24	none	0.83	1.21	0.49	0.57	0.68	0.93	0.59	0.58
14	stringent	none	24	full	0.85	1.01	0.42	0.57	0.75	1.09	0.45	0.69
15	stringent	none	6	none	0.84	1.43	0.53	0.54	0.67	0.74	0.67	0.41
16	stringent	none	6	full	0.85	0.99	0.43	0.58	0.78	0.98	0.44	0.65
17	stringent	none	0	none	0.55	1.79	0.49	0.5	0.26	1.16	1	0.22
18	stringent	none	0	full	0.65	1.02	0.38	0.5	0.46	1.47	0.52	0.21
19	medium	medium	24	none	0.91	1.05	0.41	0.45	0.91	1.03	0.43	0.57
20	medium	medium	24	full	0.95	0.99	0.37	0.45	0.94	1.06	0.41	0.56
21	medium	medium	6	none	0.91	1.49	0.45	0.43	0.9	0.9	0.46	0.39
22	medium	medium	6	full	0.95	1.05	0.38	0.45	0.93	1.03	0.41	0.51
23	medium	medium	0	none	0.53	1.49	0.43	0.44	0.57	1.19	0.56	0.16
24	medium	medium	0	full	0.66	1.04	0.35	0.44	0.57	1.51	0.45	0.15
25	medium	complete	24	none	0.91	1.06	0.41	0.46	0.91	1.02	0.43	0.57
26	medium	complete	24	full	0.95	0.99	0.36	0.46	0.93	0.94	0.4	0.56

27	medium	complete	6	none	0.9	1.46	0.44	0.44	0.9	0.91	0.47	0.38
28	medium	complete	6	full	0.94	1.12	0.37	0.46	0.93	1.06	0.41	0.51
29	medium	complete	0	none	0.66	1.66	0.43	0.43	0.56	1.25	0.56	0.18
30	medium	complete	0	full	0.59	0.91	0.35	0.43	0.57	1.45	0.47	0.15
31	medium	none	24	none	0.82	1.14	0.52	0.54	0.68	0.97	0.62	0.55
32	medium	none	24	full	0.84	0.97	0.44	0.57	0.75	1.11	0.47	0.68
33	medium	none	6	none	0.83	1.32	0.55	0.52	0.67	0.71	0.71	0.38
34	medium	none	6	full	0.85	1	0.45	0.58	0.76	1	0.46	0.62
35	medium	none	0	none	0.5	1.55	0.51	0.48	0.26	1.19	1	0.22
36	medium	none	0	full	0.58	0.96	0.41	0.48	0.46	1.46	0.53	0.21
37	none	medium	24	none	0.9	1.06	0.46	0.45	0.87	1.72	0.53	0.5
38	none	medium	24	full	0.95	1.05	0.39	0.45	0.93	1.13	0.42	0.56
39	none	medium	6	none	0.87	2.63	0.54	0.39	0.86	1.45	0.57	0.33
40	none	medium	6	full	0.94	0.98	0.41	0.45	0.93	1.08	0.44	0.51
41	none	medium	0	none	0.46	2.21	0.53	0.42	0.55	1.31	0.66	0.17
42	none	medium	0	full	0.59	0.92	0.38	0.43	0.57	1.51	0.46	0.15
43	none	complete	24	none	0.89	1.07	0.43	0.46	0.86	1.64	0.53	0.5
44	none	complete	24	full	0.94	1.04	0.37	0.46	0.93	1.06	0.42	0.56
45	none	complete	6	none	0.86	2.51	0.52	0.39	0.86	1.38	0.58	0.35
46	none	complete	6	full	0.94	1.03	0.39	0.46	0.92	1.07	0.43	0.5
47	none	complete	0	none	0.44	2.29	0.52	0.42	0.54	1.36	0.66	0.18
48	none	complete	0	full	0.55	0.92	0.37	0.41	0.57	1.51	0.48	0.16
49	none	none	24	none	0.76	1.47	0.65	0.44	0.42	1.5	0.98	0.36
50	none	none	24	full	0.84	0.99	0.44	0.58	0.74	1.02	0.5	0.66
51	none	none	6	none	0.76	1.99	0.69	0.43	0.53	0.89	0.91	0.32
52	none	none	6	full	0.83	0.95	0.45	0.57	0.74	0.92	0.48	0.58
53	none	none	0	none	0.39	1.43	0.92	0.39	0.26	1.16	1	0.22
54	none	none	0	full	0.58	0.93	0.44	0.47	0.46	1.43	0.54	0.2

'Stringent' sample QC comprised exclusion of outliers for LRR mean, LRR DLRS and GC-Wave at the lower and upper bound thresholds, stringent exclusion of chromosomal aberrations, exclusion of plates with more than 30% samples excluded on these former criteria. 'Medium' sample QC used 3.5 standard deviations as the threshold for LRR Mean, DLRS and GC-Wave, used a more relaxed threshold for chromosomal aberrations, and excluded plates with more than 40% of samples excluded. 'Complete' SNP-QC involved exclusion of samples for low call rate, exclusion of SNPs with call rate below 99%, exclusion of SNPs violating a HWE threshold of 10^-4, exclusion of SNPs with significant differences in missingness or HWE between cohorts, and exclusion of samples with extreme heterozygosity. 'Medium' SNP-QC used a

SNP call rate of 95%, a HWE threshold of 10^-10, and did not apply the exclusion for cohort differences.

There are several contexts to consider for expected sensitivity and specificity. Firstly, as a ’gold-standard’, results from the birdsuite validation [3] showed sensitivity ranging from ~30% to ~60% in the range from 6-20 probes per CNV, and this significantly outperformed other methods from 2008. Another group found roughly 67% specificity with 50% sensitivity using Illumina SNP arrays, but this included common CNVs[21].
Notably, if sensitivity varies a lot, then the ’rare’ frequency cutoff could artificially reduce sensitivity. Detection of CNVs at a rate below the true population frequency could readily occur in the present study, or may have been the case in some DGV studies. It may be many CNVs found previously are so rare that we are unlikely to find them. The expected distribution for the population frequency of CNVs present within an individual is thought to be an exponentially decreasing function, implying that within an individual most CNVs are relatively common, but within a population most CNVs are rare, including CNVs unique to certain persons. Furthermore, after a recent update in the DGV it is very difficult to filter studies by the ethnicities included, so some DGV CNVs could be specific to non-european populations, making them difficult to detect in the current study of European samples. Lastly, QC for CNVs is still being developed so it is highly likely that the DGV itself contains many false-positive and false-negatives. Given the performance of birdsuite, 60% was the absolute upper level of sensitivity expected, although considering the limitations discussed we thought it more likely to achieve half this accuracy.

Supplementary Table 6. Descriptive listing of de novo rDELs from T1D affected offspring

CNV location					Sample, age at:				
locus	start (bp)	end (bp)	width (bp)	genes intersected	diagnosis	blood draw	sex	HLA DRB1 Risk*****	SNP sites
1q23.3	159,766,754	159,778,387	11,634	*FCGR3A*	8	12	F	HIGH	111
1q23.3	159,824,152	159,885,964	60,812	*FCGR3A*	13	33	M		44
1q21.3	150,801,645	150,848,998	47,354	*LCE3E;LCE3D;LCE3C*	5	12	M*	.	43
1p22.1	92,351,794	92,357,107	5,314	*BTBD8*	2	14	F	HIGH	14
1q31.3	193,488,929	193,508,304	19,376	intergenic, between *EEF1AL12* and *KCNT2*	30	39	F		6
2p22.3	33,546,551	33,549,327	2,777	*RASGRP3*	6	18	M**		12
2q36.3-37.3	227,561,401	242,669,396	15,107,996	*RHBDD1* + 146 others***	11	17	M	HIGH	1750
2q13	111,420,610	112,375,250	954,641	*ANAPC1;MERTK;ACOXL;BCL2L11*	21	32	F		26
2q13	111,168,287	112,805,738	1,637,452	*ACOXL;BCL2L11;ANAPC1;MERTK;TMEM87B;FBLN7;ZC3H8;ZC3H6*	5	25	F		41
3p24.3	21,285,786	21,503,924	218,139	*ZNF385D*	10	17	F	HIGH	8
3p21.31	46,958,534	47,883,819	925,286	*CCDC12;SETD2;KIF9;SNORD13;KLHL18;PTPN23;SCAP;C3orf75;CSPG5;SMARCC1;DHX30;MIRN1226;MAP4*	11	17	M		28
5q15	96,235,252	96,235,711	460	intergenic, between *ERAP1* and *ERAP2*	18	22	F	HIGH	6
5q11.2-12.3	55,287,688	64,520,558	9,232,871	*IL6ST*+ 31 others****	16	17	F	HIGH	256
6p21.33	30,024,484	30,028,142	3,659	intergenic, between *HCG4P6* and *HCG9*	2	12	M	HIGH	10
6p21.33	31,407,142	31,409,966	2,825	*HLA-C*	6	14	F		10
16p11.2†	28,730,320	28,933,479	203,160	*ATXN2L;TUFM;SH2B1;ATP2A1;RABEP2;CD19;NFATC2IP;SPNS1;LAT*	7	17	F	HIGH	217
16p11.2†	28,730,320	28,933,479	203,160	*ATXN2L;TUFM;SH2B1;ATP2A1;RABEP2;CD19;NFATC2IP;SPNS1;LAT*	6	15	F	HIGH	217
16p13.3	6,061,558	6,068,521	6,964	intergenic, between *FAM86B2* and *C16orf68*	3	7	M	HIGH	6

LEGEND:

† this CNV lies within a known T1D region

... Legend continues over page ...

LEGEND continued:

* this is the only T1D sample in the table that does not have an affected sibling (recruitment targeted families with at least two T1D children)

** this is the only sample in the table with an affected parent - was the father

*** The remaining 146 genes were:
COL4A4;COL4A3;MFF;TM4SF20;AGFG1;C2orf83;SLC19A3;CCL20;WDR69;SPHKAP;PID1;DNER;TRIP12;FBXO36;SLC16A14;SP110;SP140;SP140L;SP100;CAB39;ITM2C;GPR55;SPATA3;C2orf72;PSMD1;HTR2B;ARMC9;B3GNT7;NCL;SNORA75;SNORD20;SNORD82;C2orf52;NMUR1;C2orf57;PTMAP4;PTMA;PDE6D;COPS7B;MIRN1471;NPPC;FAM6A;DIS3L2;MIRN562;ALPP;ECEL1P2;ALPPL2;ALPI;ECEL1;CHRND;CHRNG;TIGD1;EIF4E2;EFHD1;GIGYF2;KCNJ13;C2orf82;NGEF;NEU2;INPP5D;ATG16L1;SCARNA5;SCARNA6;SAG;DGKD;USP40;UGT1A12P;UGT1A11P;UGT1A9;UGT1A3;UGT1A5;UGT1A6;UGT1A1;UGT1A8;UGT1A4;UGT1A10;UGT1A7;UGT1A13P;DNAJB3;UGT1A2P;HEATR7B1;HJURP;TRPM8;SPP2;ARL4C;SH3BP4;AGAP1;GBX2;ASB18;IQCA1;CXCR7;COPS8;COL6A3;MLPH;PRLH;RAB17;LRRFIP1;RBM44;RAMP1;UBE2F;SCLY;ESPNL;KLHL30;FAM132B;ILKAP;HES6;PER2;TRAF3IP1;ASB1;HDAC4;NDUFA10;OR6B2;OR6B3;OR9S24P;MYEOV2;OTOS;GPC1;MIRN149;ANKMY1;DUSP28;RNPEPL1;CAPN10;GPR35;AQP12B;AQP12A;KIF1A;AGXT;C2orf54;SNED1;MTERFD2;PASK;PPP1R7;ANO7;HDLBP;SEPT2;FARP2;STK25;BOK;THAP4;ATG4B;DTYMK;ING5;D2HGDH;GAL3ST2;NEU4;PDCD1;C2orf85

**** The remaining 31 genes were:
ANKRD55;MAP3K1;C5orf35;MIER3;GPBP1;ACTBL2;PLK2;GAPT;RAB3C;PDE4D;MIRN582;DEPDC1B;ELOVL7;ERCC8;NDUFAF2;C5orf43;FAM133B;RPL3P6;ZSWIM6;KIF2A;DIMT1L;IPO11;LRRC70;ISCA1L;HTR1A;RNF180;RGS7BP;FAM159B;SFRS12IP1;SDCCAG10;ADAMTS6

***** HLA DRB1 risk was high if alleles 1 and 2 were *03/*04, *03/*03 or *04/*04

Supplementary Table 7. Summary of genes for which we performed qPCR validation tests on samples for which DNA was available.

**GENE**	**Case-Control Study**		**Family Cohort**		**Total Tested CNVs**	**qPCR Validated CNVs**
	**Case CNVs**	**Control CNVs**	**AFF CNVs**	**UNAFF CNVs**		
PKIA[#]	6 DELs	1 DEL	-	-	5 DELs	5 DELs
SH2B1	7 DELs	3 DELS<br>6 DUPs	6 DELs	0	16 DELs<br>5 DUPs	16 DELs<br>5 DUPs
*MKKS**	-	-	5 DELs<br>1 DUP	0	5 DELs<br>1 DUP	5 DELs<br>1 DUP
				Total CNVs	32	32

Note. DELs = deletions; AFF = affected siblings and/or parents; UNAFF = unaffected siblings and/or parents. * *MKKS* DELs spanned 3 SNPs and were tested to verify if 3-SNP DELs identified in the five offspring were true CNV calls.

Supplementary Table 8. Comparison of genome coverage between ImmunoChip and the 550k Illumina GWAS array. Each cell states the percentage of the genome covered with at least a 6 SNP density per potential window-size, as this was the optimal size determined using the process described in Supplementary Methods 3. It can be seen that ImmunoChip has much better coverage for small CNVs in T1D and immune regions, whereas the GWAS chip has better genome-wide coverage for CNVs 10 kb or larger. Immune regions are the regions identified by consortia for 12 autoimmune diseases as described in the Materials and Methods section regarding 'platform'. T1D regions are a subset related to T1D, as catalogued at immunobase.org (66).

	**ImmunoChip %**			**HumanHap550K %**		
**Window size**	*T1D regions*	*Immune regions*	*Human genome*	*T1D regions*	*Immune regions*	*Human genome*
≥ 5 kb	83.4	85.1	2.0	1.2	1.0	1.5
≥ 10 kb	95.0	94.5	2.5	7.9	7.2	10.4
≥ 20 kb	97.1	96.3	2.9	27.1	28.9	38.5
≥ 50 kb	97.4	96.9	4.9	61.8	69.2	79.8
≥ 100 kb	99.6	98.8	10.5	90.2	89.7	89.6
≥ 200 kb	99.6	100.0	27.2	93.6	94.3	91.4
≥ 400 kb	100.0	100.0	58.7	99.6	97.5	92.2
≥ 1 Mb	100.0	100.0	88.7	100.0	100.0	93.0
≥ 3 Mb	100.0	100.0	92.9	100.0	100.0	93.5
